# Supplementary material for: Origin and maintenance of large ribosomal RNA gene repeat size in mammals
Source: Genetics. 2024 Jul 24;228(1):iyae121. doi: 10.1093/genetics/iyae121 (PMC11373518; doi:10.1093/genetics/iyae121)
Supplement: iyae121_Supplementary_Data [file iyae121_supplementary_data.zip › Figure_S1_GENETICS-2024-307168.pdf]

A

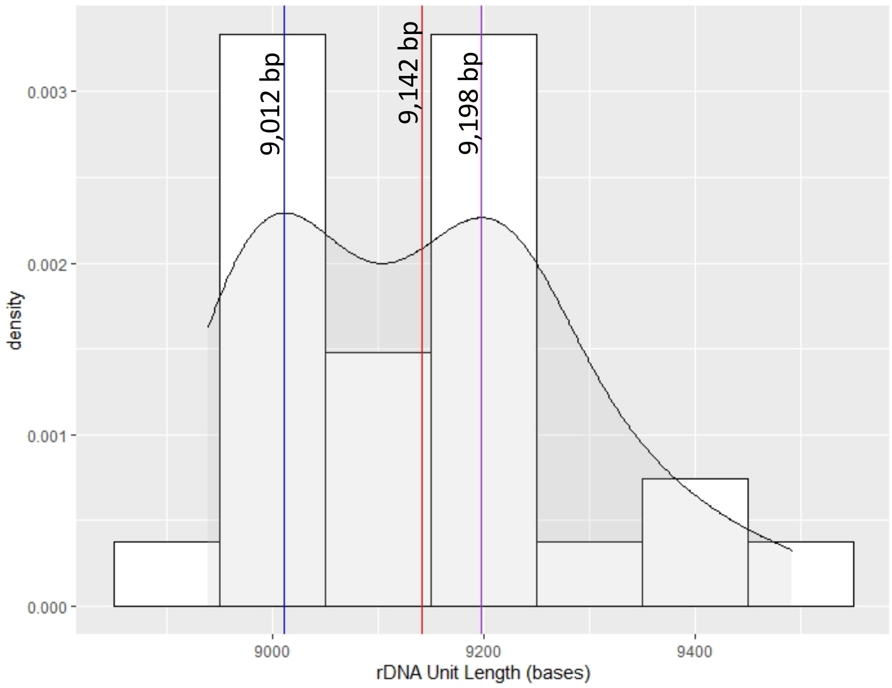
9,012 bp
9,142 bp
9,198 bp
density
0.003
0.002
0.001
0.000
9000
9200
9400
rDNA Unit Length (bases)

B

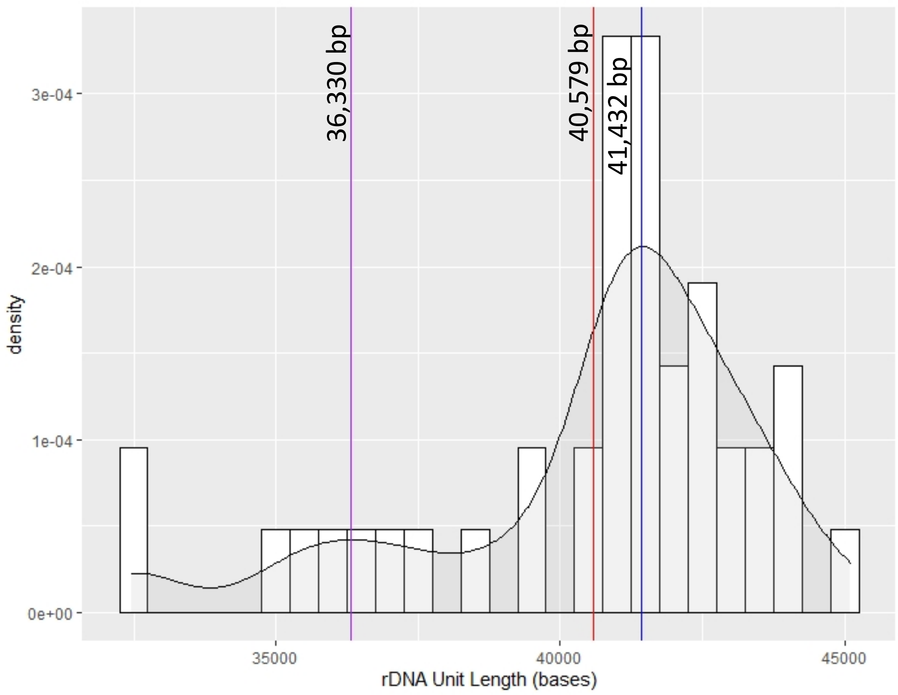
36,330 bp
40,579 bp
41,432 bp
density
3e-04
2e-04
1e-04
0e+00
35000
40000
45000
rDNA Unit Length (bases)

C

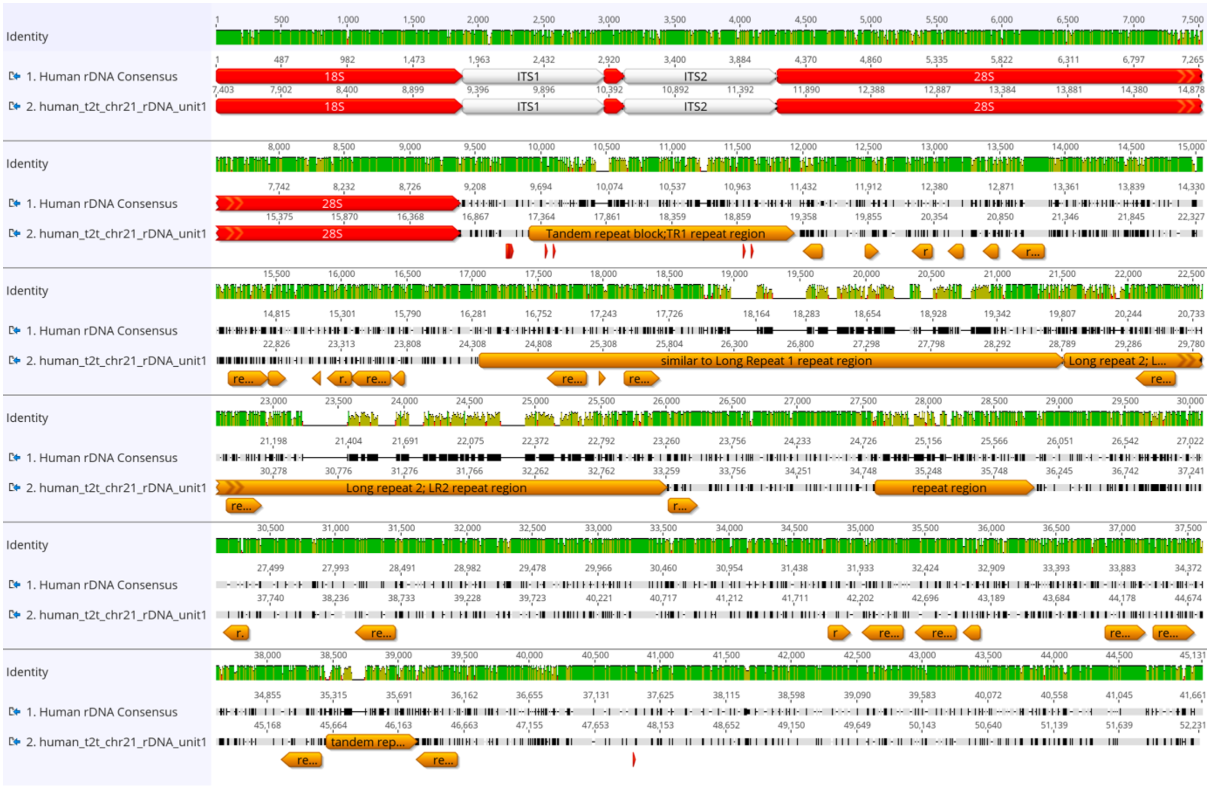

Figure S1. ONT sequence read BLAST analysis accurately distinguishes 'normal' and 'long' rDNA units. Density plots and histograms showing the distribution of total rDNA unit length measured using ONT reads from (**A**) *Saccharomyces cerevisiae* with a bin size of 100 bp; and (**B**) human with a bin size of 500 bp. Mean rDNA unit size (red vertical line) and the centres of peak densities (blue and purple vertical lines) are indicated. The different peaks may represent copy number variation of repeats within the IGS region. (**C**) Geneious output of an alignment of the human consensus sequence with a chromosome 21 rDNA unit from the human telomere-to-telomere genome assembly. Matching nucleotides are indicated in green above the sequences, and rDNA annotations are shown for the two sequences.
