# Supplementary material for: Origin and maintenance of large ribosomal RNA gene repeat size in mammals
Source: Genetics. 2024 Jul 24;228(1):iyae121. doi: 10.1093/genetics/iyae121 (PMC11373518; doi:10.1093/genetics/iyae121)
Supplement: iyae121_Supplementary_Data [file iyae121_supplementary_data.zip › Figure_S10_GENETICS-2024-307168.pdf]

A

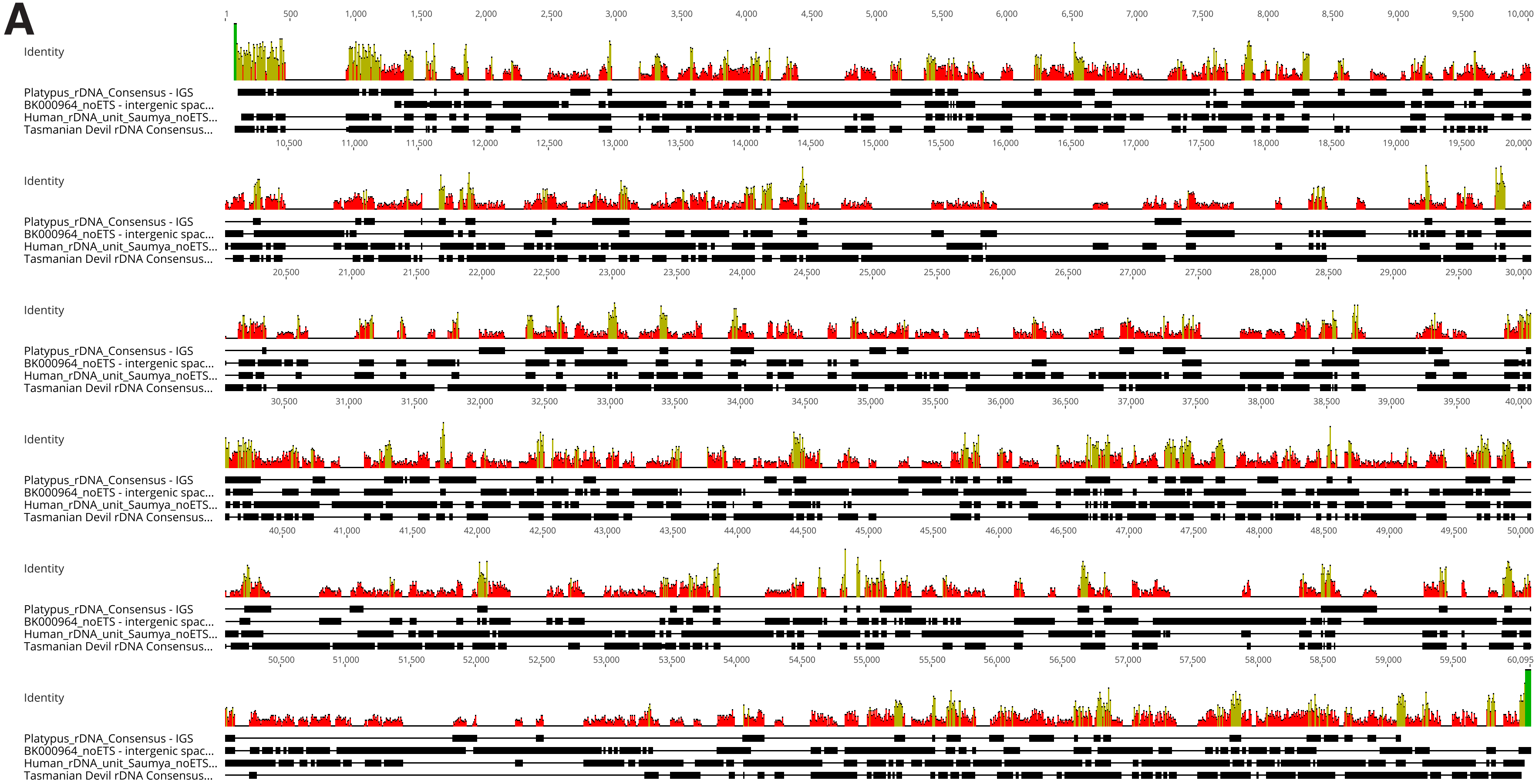

B

	Platypus_r...	BK000964_...	Human_rD...	Tasmanian ...
Platypus_rDNA_Consen...		6.3%	8.9%	8.2%
BK000964_noETS - inter...	6.3%		17.2%	14.4%
Human_rDNA_unit_Sau...	8.9%	17.2%		22.9%
Tasmanian Devil rDNA ...	8.2%	14.4%	22.9%	

Figure S10. Mammalian IGS sequences are essentially unalignable. A. Geneious output of an alignment of platypus, mouse, human and Tasmanian devil IGS sequences (in the order listed). **B.** Pairwise distance matrix from the alignment shown in **A**.
