# Supplementary material for: Origin and maintenance of large ribosomal RNA gene repeat size in mammals
Source: Genetics. 2024 Jul 24;228(1):iyae121. doi: 10.1093/genetics/iyae121 (PMC11373518; doi:10.1093/genetics/iyae121)
Supplement: iyae121_Supplementary_Data [file iyae121_supplementary_data.zip › Figure_S2_GENETICS-2024-307168.pdf]

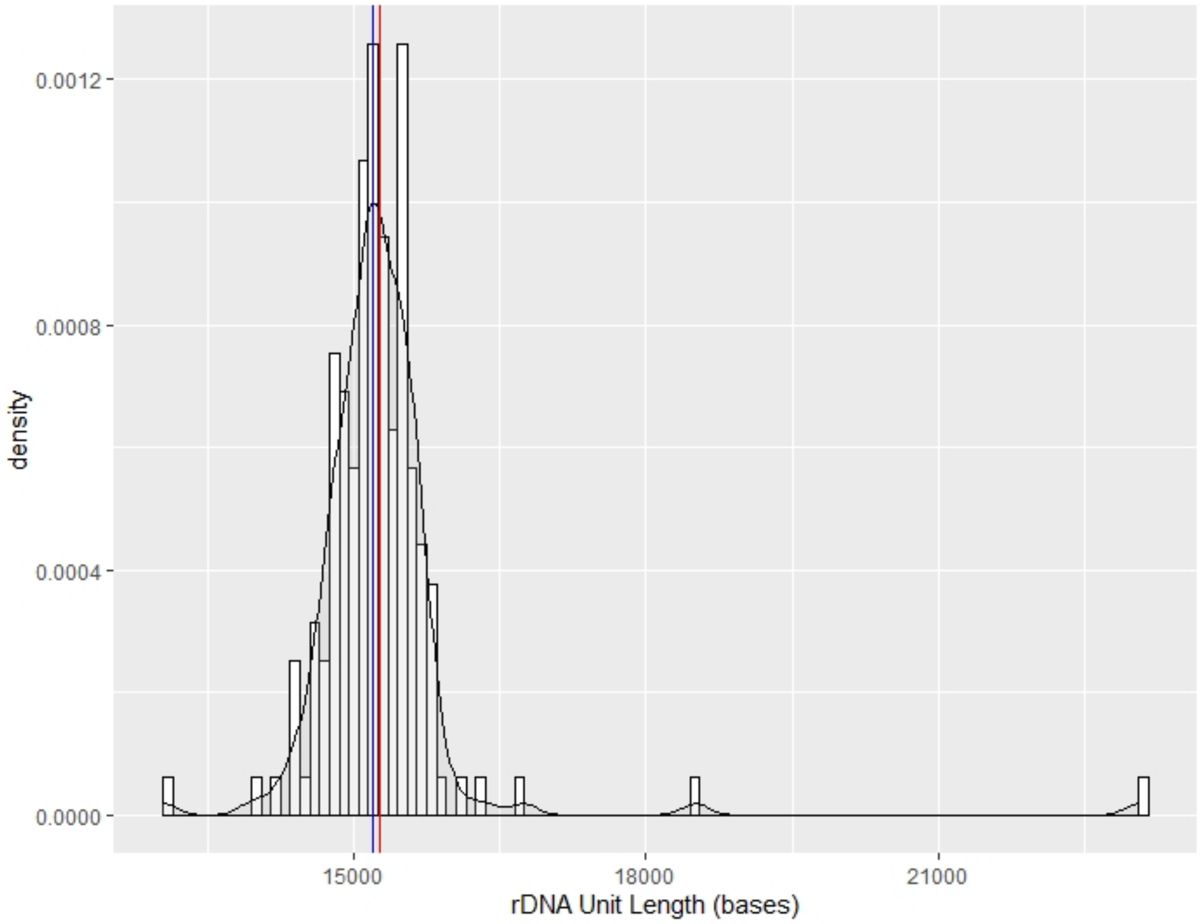

***Pseudonaja textilis* (brown snake) rDNA unit size from ONT sequence reads.** Mean rDNA unit size (15,627 bp, red vertical line) and the centre of the peak of the density curve (15,602 bp, blue vertical line) are indicated.

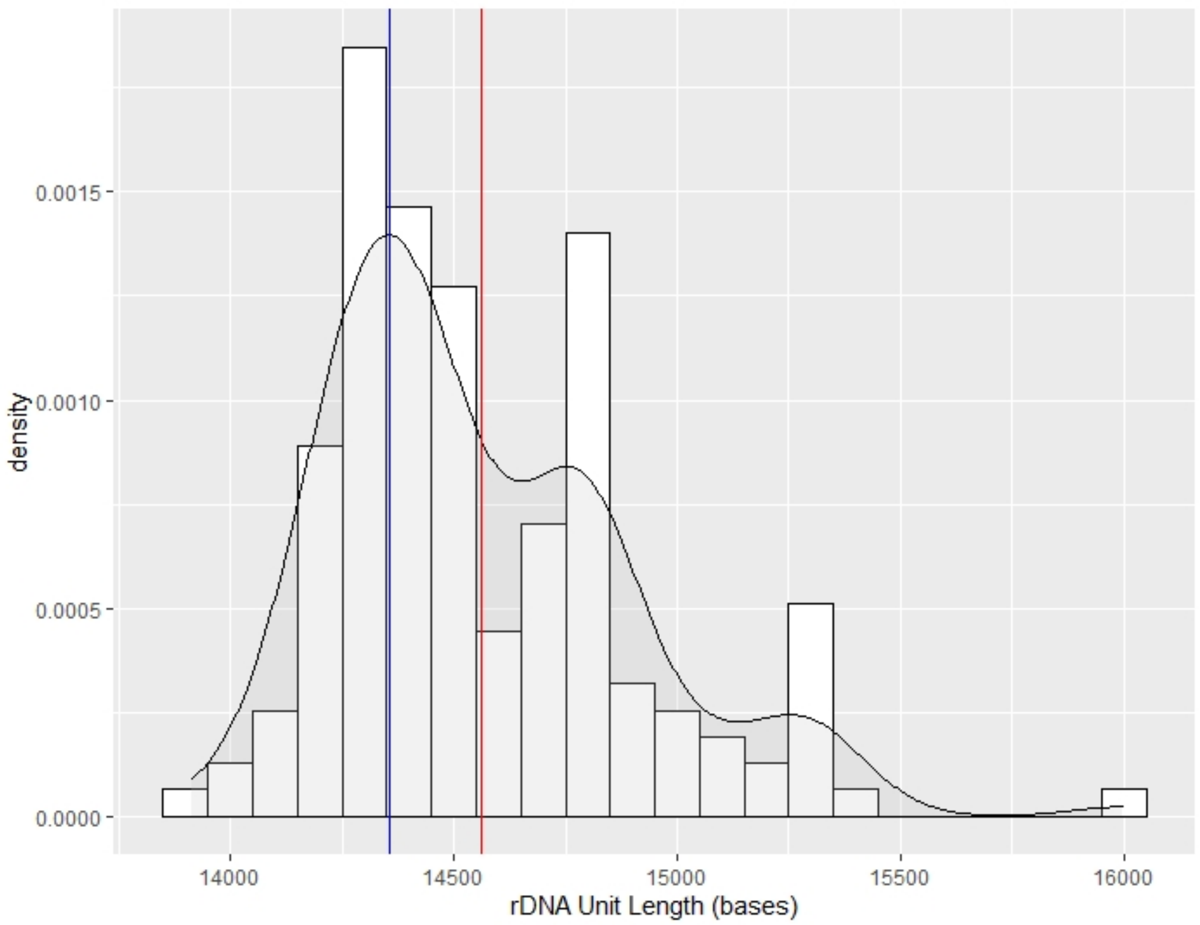

***Notechis scutatus* (tiger snake) rDNA unit size from ONT sequence reads**. Mean rDNA unit size (14,562 bp, red vertical line) and the centre of the peak of the density curve (14,354 bp, blue vertical line) are indicated.

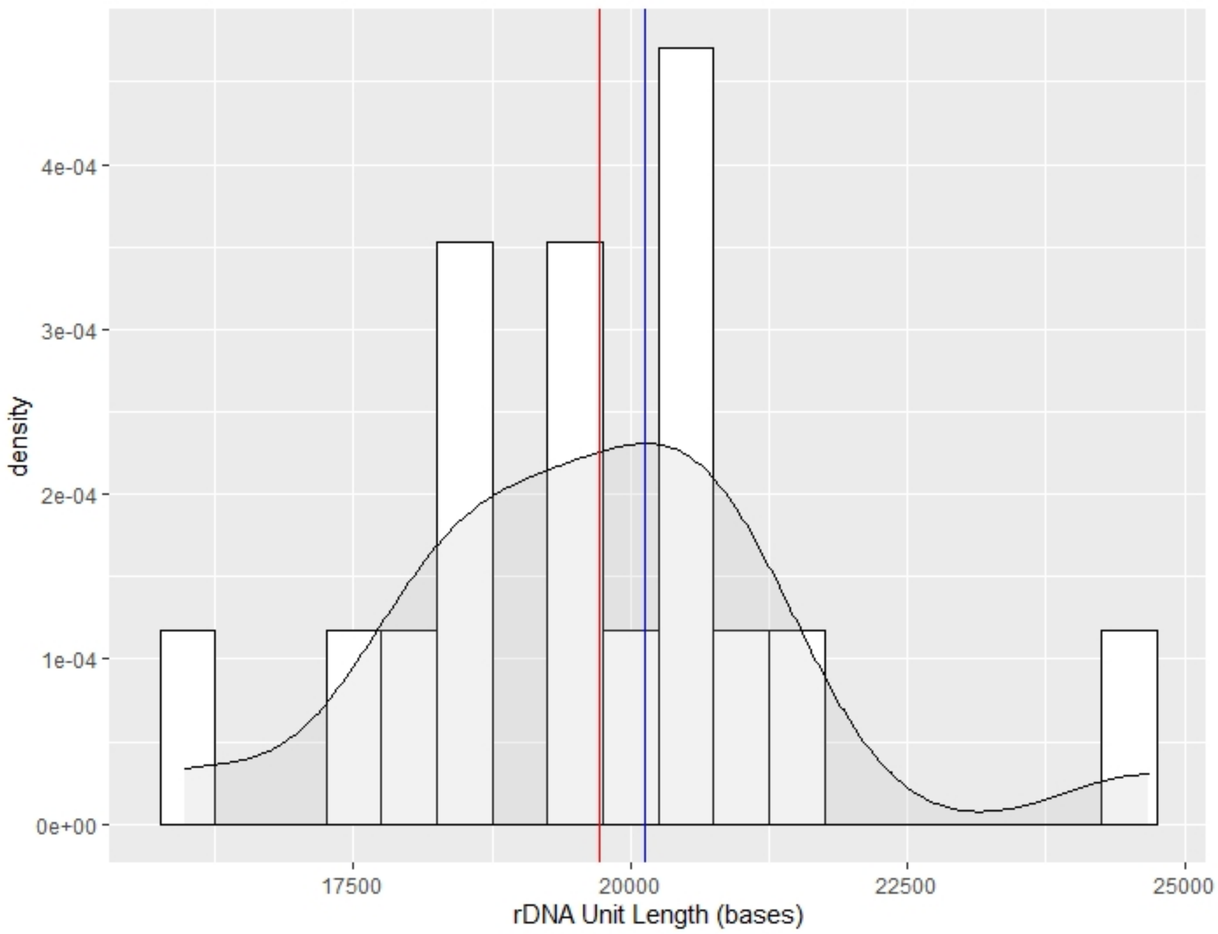

Sphenodon punctatus **(Tuatara) rDNA unit size from ONT sequence reads.** Mean rDNA unit size (19,721 bp, red vertical line) and the centre of the peak of the density curve (20,134 bp, blue vertical line) are indicated.

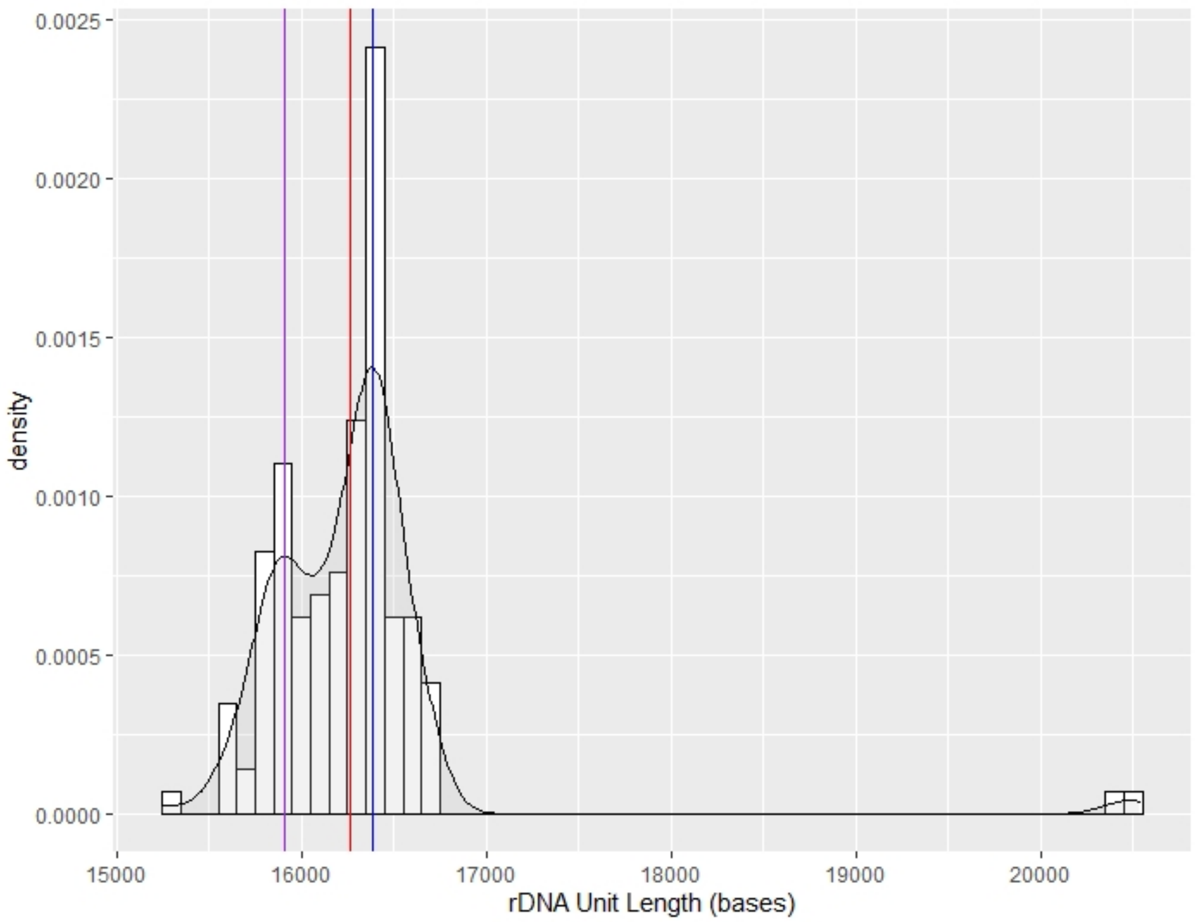

***Melanerpes aurifrons* (golden-fronted woodpecker) rDNA unit size from ONT sequence reads.** Mean rDNA unit size (19,721 bp, red vertical line) and the centres of the density peaks (20,134 bp, blue vertical line; 15,907 bp, purple vertical line) are indicated.

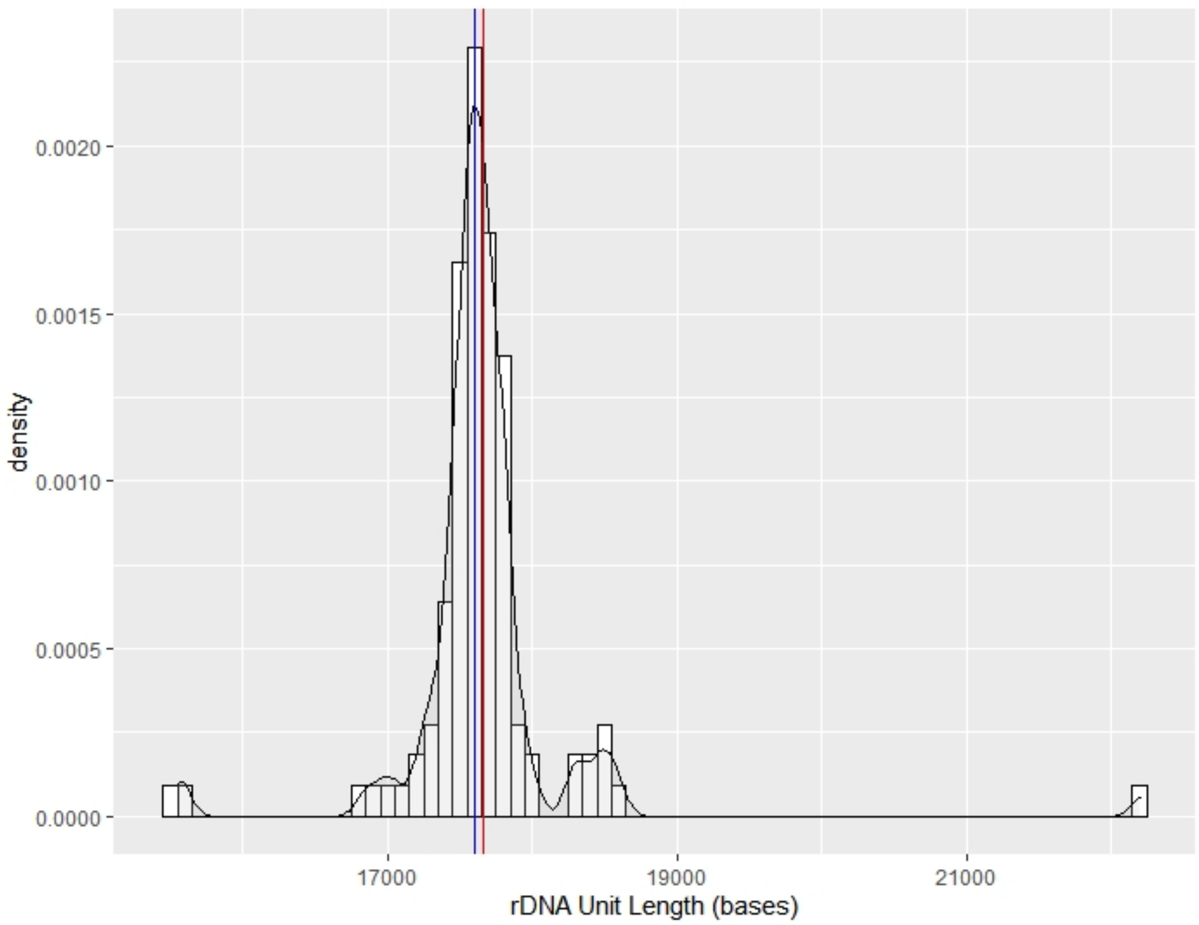

***Notiomystis cincta* (stitchbird hihi) rDNA unit size from ONT sequence reads.** Mean rDNA unit size (17,685 bp, red vertical line) and the centre of the peak of the density curve (17,601 bp, blue vertical line) are indicated.

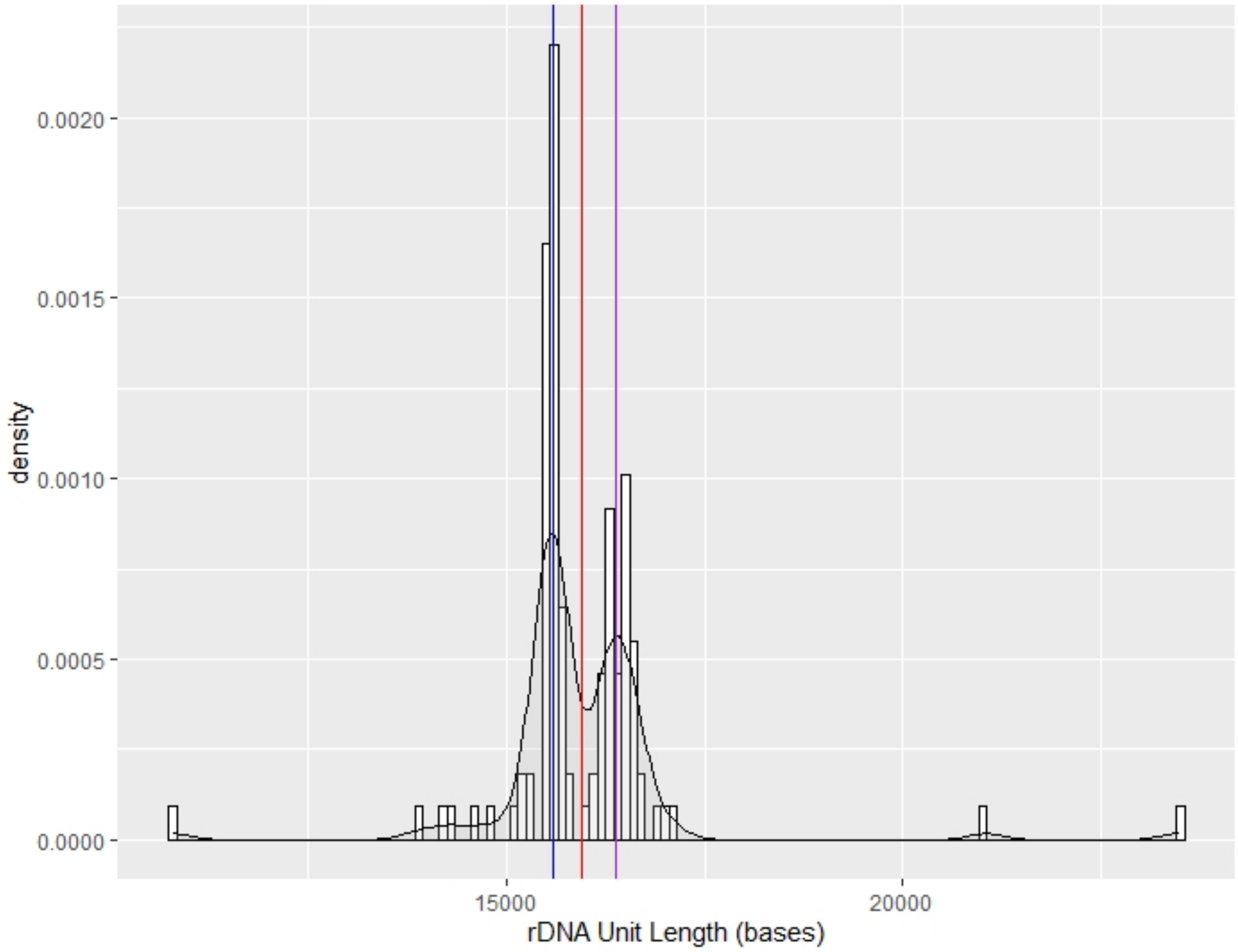

***Rhinella marina* (cane toad) rDNA unit size from ONT reads.** Mean rDNA unit size (15,942 bp, red vertical line) and the centres of the density peaks (15,592 bases, blue vertical line; 16,382 bases, purple vertical line) are indicated.

Figure S2. Determination of rDNA unit sizes from ONT sequence reads. Measured rDNA unit sizes are plotted as density plots and histograms, all with a bin size of 100 bp. Panels show data for *Pseudonaja textilis* (brown snake), *Notechis scutatus* (tiger snake), *Sphenodon punctatus* (Tuatara), *Melanerpes aurifrons* (golden-fronted woodpecker), *Notiomystis cincta* (stitchbird hihi), *Malaclemys terrapin* (diamondback terrapin), *Rhinella marina* (cane toad). Refer to each panel for details. See **Figure S3** for the equivalent *Sarcophilus harrisii* (Tasmanian devil) data.
