# Supplementary material for: Origin and maintenance of large ribosomal RNA gene repeat size in mammals
Source: Genetics. 2024 Jul 24;228(1):iyae121. doi: 10.1093/genetics/iyae121 (PMC11373518; doi:10.1093/genetics/iyae121)
Supplement: iyae121_Supplementary_Data [file iyae121_supplementary_data.zip › Figure_S3_GENETICS-2024-307168.pdf]

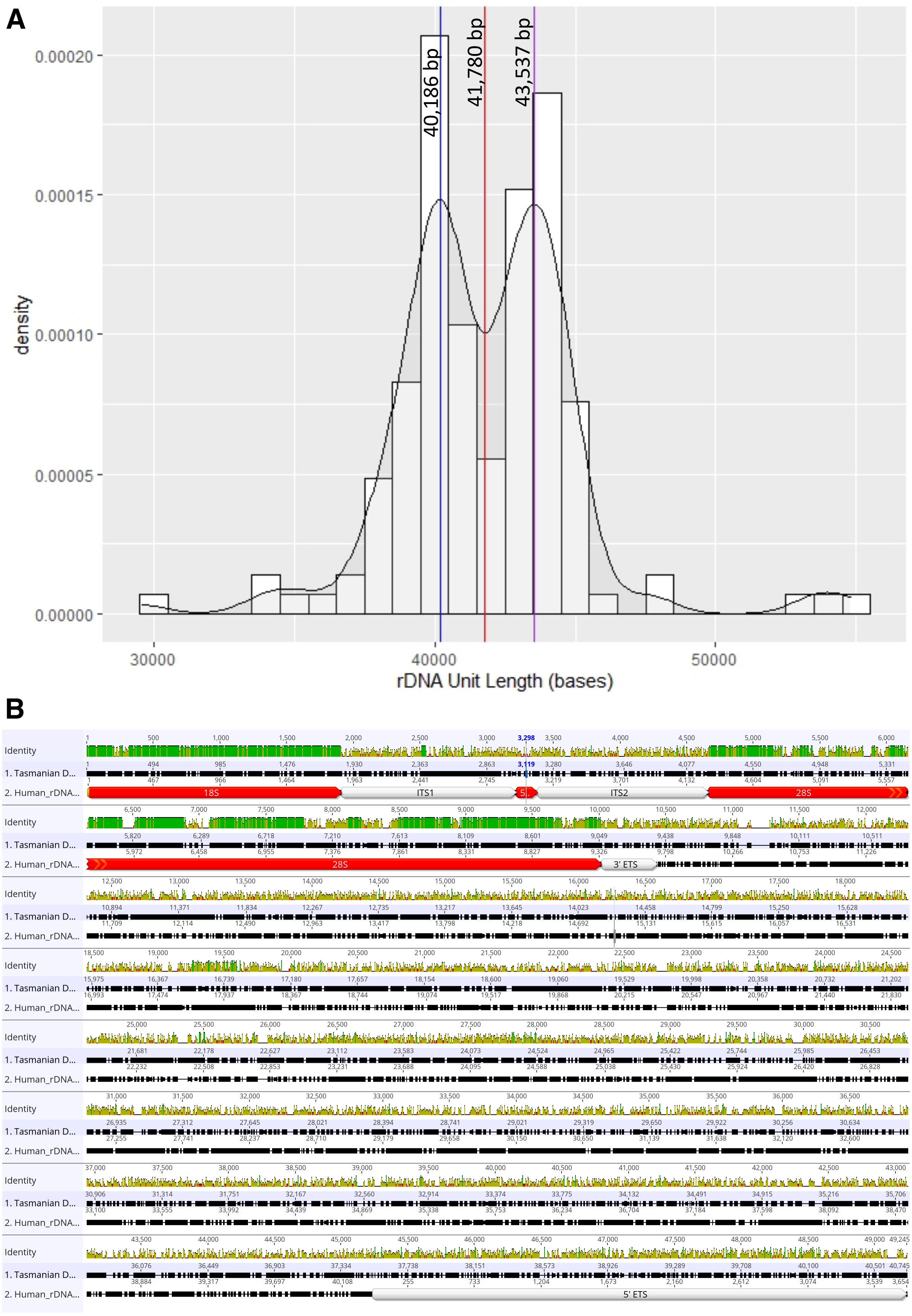
A
B
40,186 bp
41,780 bp
43,537 bp
density
0.00020
0.00015
0.00010
0.00005
0.00000
30000
40000
50000
rDNA Unit Length (bases)
Identity
1. Tasmanian D...
2. Human_rDNA...
18S
ITS1
5.
ITS2
28S
3' ETS
5' ETS

Figure S3. Determination of *Sarcophilus harrisii* (Tasmanian Devil) rDNA unit size from ONT sequence reads. A. Measured rDNA unit sizes are plotted as a density plot and a histogram (with a bin size of 1,000 bp). Mean rDNA unit size (41,780 bases, red vertical line), and the centres of the density peaks (blue (40,186 bp) and purple (43,537 bp) vertical lines) are indicated. **B.** Geneious alignment of the short Tasmanian Devil rDNA unit aligned to the human rDNA unit. The lack of conservation of the 5.8S rRNA gene (second red arrow) can be seen by the lack of green high identity bars above the 5.8S annotation, while these are clearly seen for the 18S and 28S rRNA genes.
