# Supplementary material for: Origin and maintenance of large ribosomal RNA gene repeat size in mammals
Source: Genetics. 2024 Jul 24;228(1):iyae121. doi: 10.1093/genetics/iyae121 (PMC11373518; doi:10.1093/genetics/iyae121)
Supplement: iyae121_Supplementary_Data [file iyae121_supplementary_data.zip › Figure_S4_GENETICS-2024-307168.pdf]

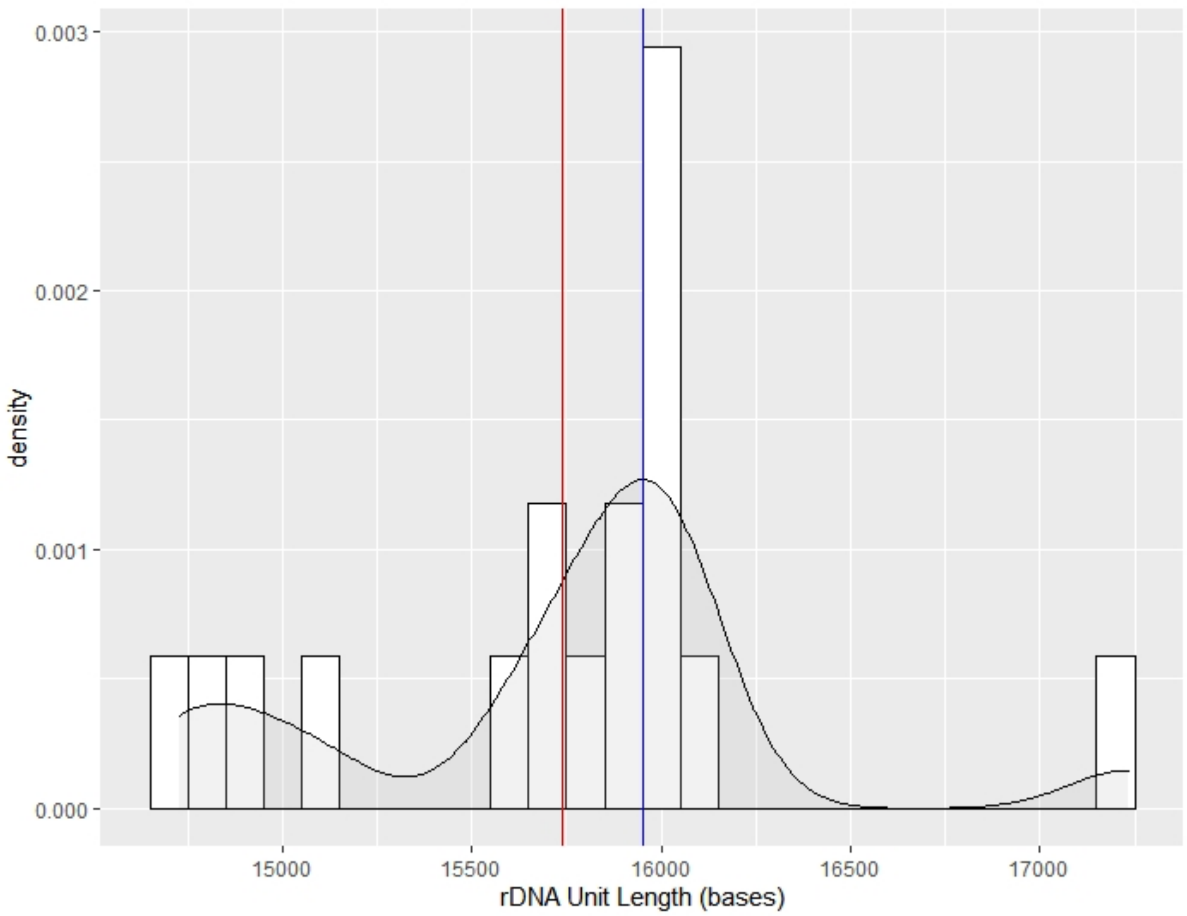


**Figure S4. Determination of rDNA unit size *Malaclemys terrapin* (Diamondback terrapin) from a PacBio assembly**. Measured rDNA unit sizes from a publicly-available *M. terrapin* assembly are plotted as density plots and histograms with a bin size of 100 bp. Mean rDNA unit size (15,740 bp, red vertical line), the centre of the peak of the density curve (15,950 bp, blue vertical line) are indicated.
