# Supplementary material for: Origin and maintenance of large ribosomal RNA gene repeat size in mammals
Source: Genetics. 2024 Jul 24;228(1):iyae121. doi: 10.1093/genetics/iyae121 (PMC11373518; doi:10.1093/genetics/iyae121)
Supplement: iyae121_Supplementary_Data [file iyae121_supplementary_data.zip › Figure_S5_GENETICS-2024-307168.pdf]

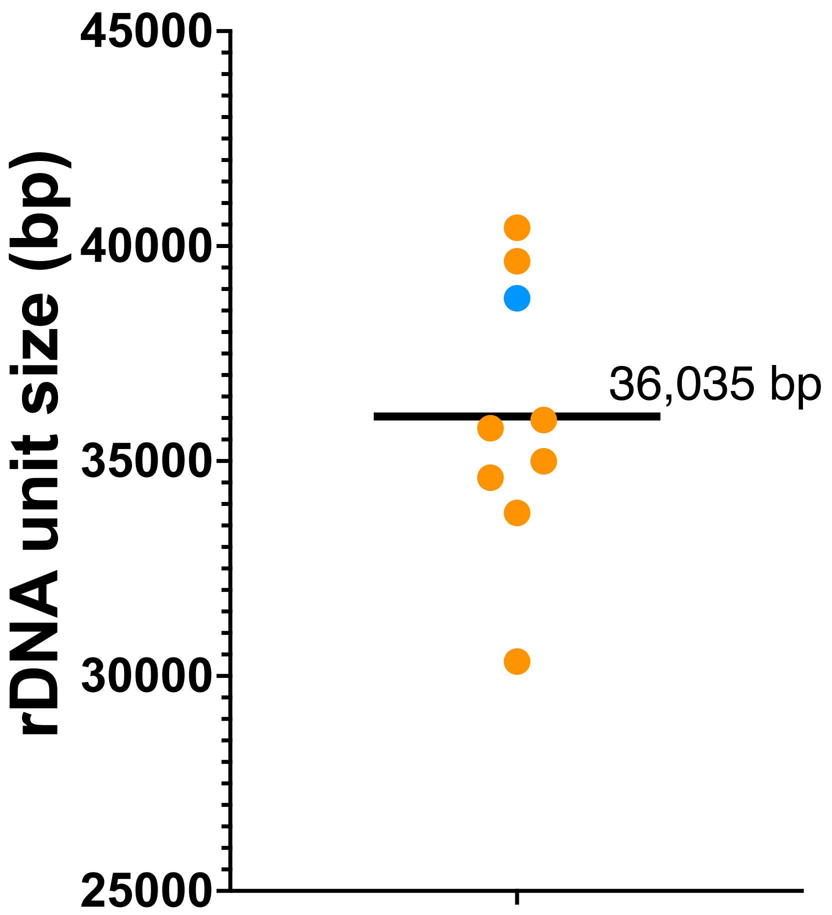

Figure S5. Platypus rDNA units are large. Sizes (in bp) of individual PacBio rDNA units are plotted. The mean size is shown.
