# Supplementary material for: Origin and maintenance of large ribosomal RNA gene repeat size in mammals
Source: Genetics. 2024 Jul 24;228(1):iyae121. doi: 10.1093/genetics/iyae121 (PMC11373518; doi:10.1093/genetics/iyae121)
Supplement: iyae121_Supplementary_Data [file iyae121_supplementary_data.zip › Figure_S6_GENETICS-2024-307168.pdf]

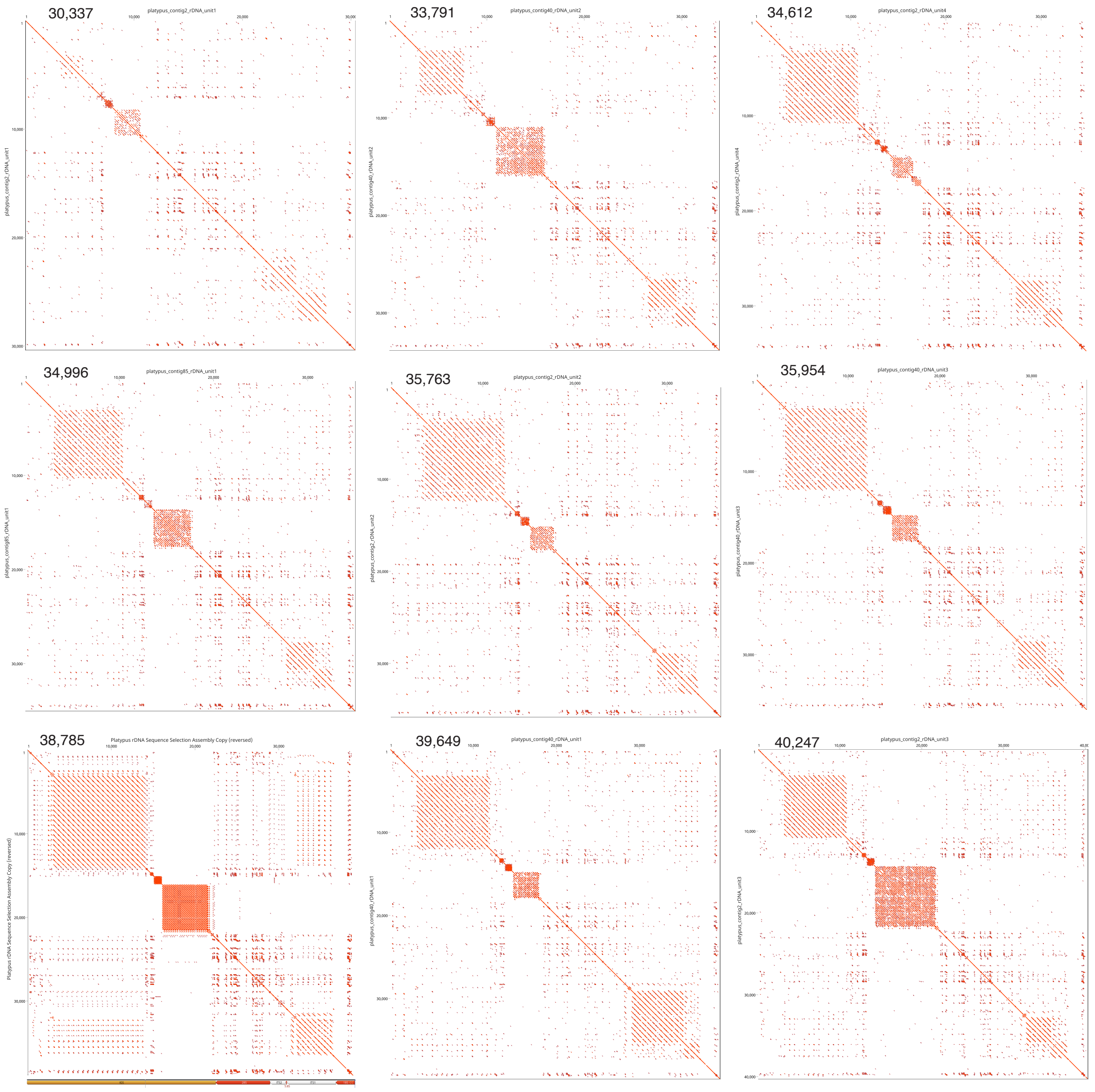

Figure S6. Dotplots of platypus rDNA units showing variation in the size of tandem sub-repeat arrays. Dotplots are of the individual platypus rDNA units from the two Pacbio assemblies. The first (Zhou et al) PacBio assembly unit is at bottom left, with an rDNA unit map across the bottom. Note the orientation is in reverse order compared to **Figure 3** and **Figure S7**, with the IGS on the left and the coding region on the right. The remaining rDNA units are from the second PacBio assembly. rDNA unit sizes are indicated above each dotplot. Red lines indicate sequence matches in an all-versus-all alignment. Sub-repeat arrays appear as red ‘squares’ around the diagonal. Dotplots were created in Geneious (v. 2020.05).
