# Supplementary material for: Origin and maintenance of large ribosomal RNA gene repeat size in mammals
Source: Genetics. 2024 Jul 24;228(1):iyae121. doi: 10.1093/genetics/iyae121 (PMC11373518; doi:10.1093/genetics/iyae121)
Supplement: iyae121_Supplementary_Data [file iyae121_supplementary_data.zip › Figure_S7_GENETICS-2024-307168.pdf]

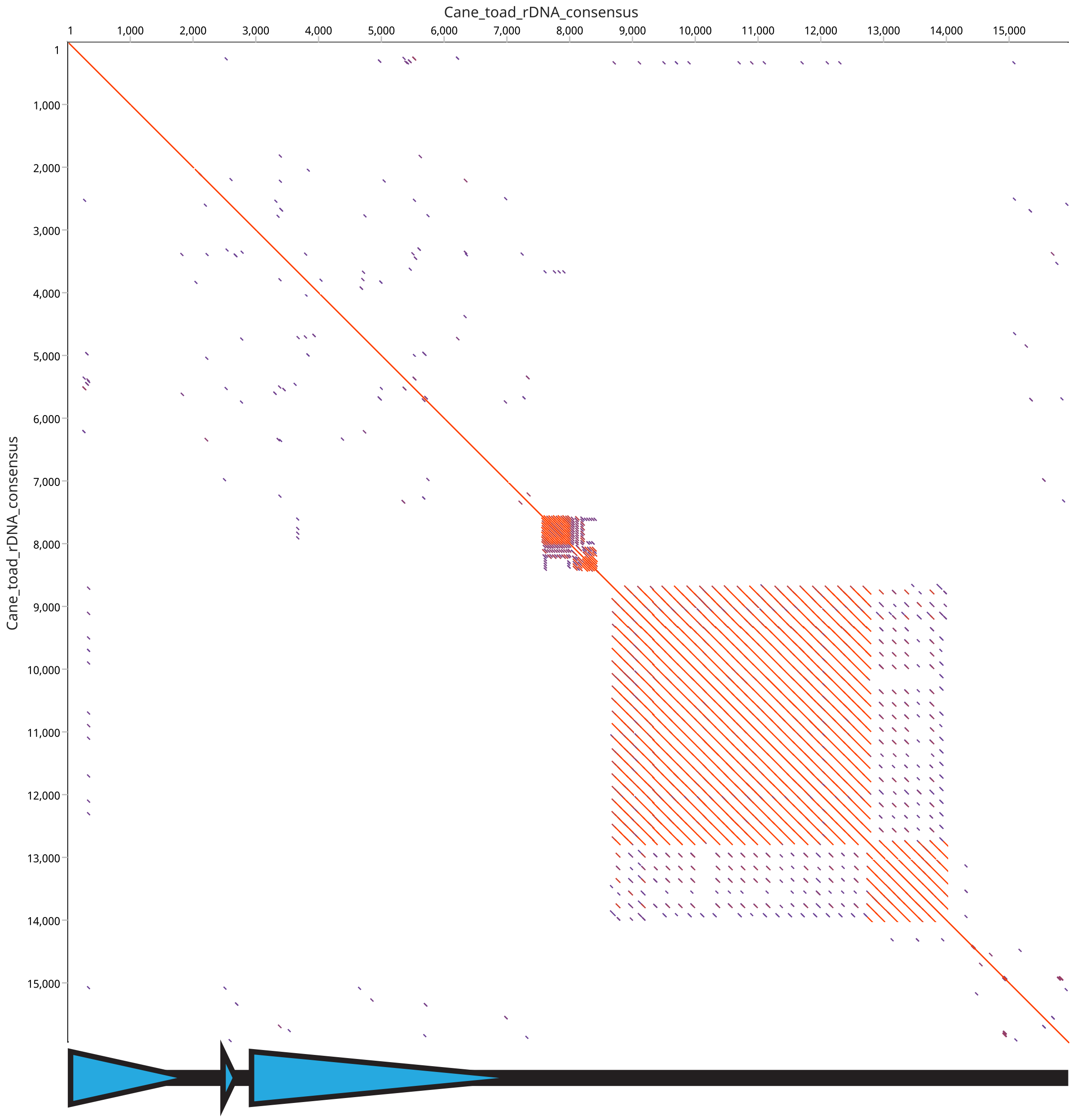
Cane_toad_rDNA_consensus
1
1,000
2,000
3,000
4,000
5,000
6,000
7,000
8,000
9,000
10,000
11,000
12,000
13,000
14,000
15,000
Cane_toad_rDNA_consensus

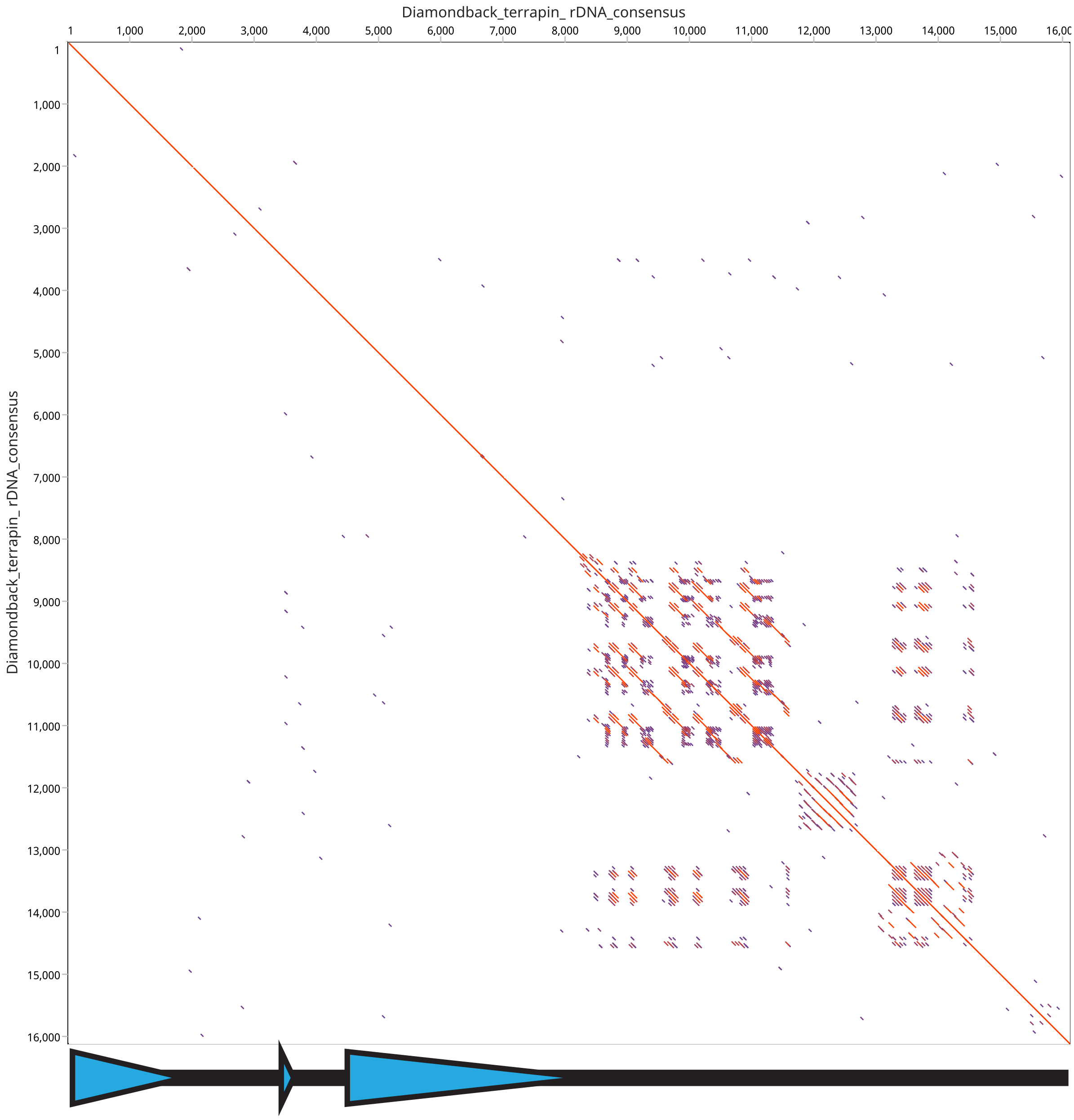

Diamondback_terrapin_ rDNA_consensus
1
1,000
2,000
3,000
4,000
5,000
6,000
7,000
8,000
9,000
10,000
11,000
12,000
13,000
14,000
15,000
16,00
Diamondback_terrapin_ rDNA_consensus
1
1,000
2,000
3,000
4,000
5,000
6,000
7,000
8,000
9,000
10,000
11,000
12,000
13,000
14,000
15,000
16,000

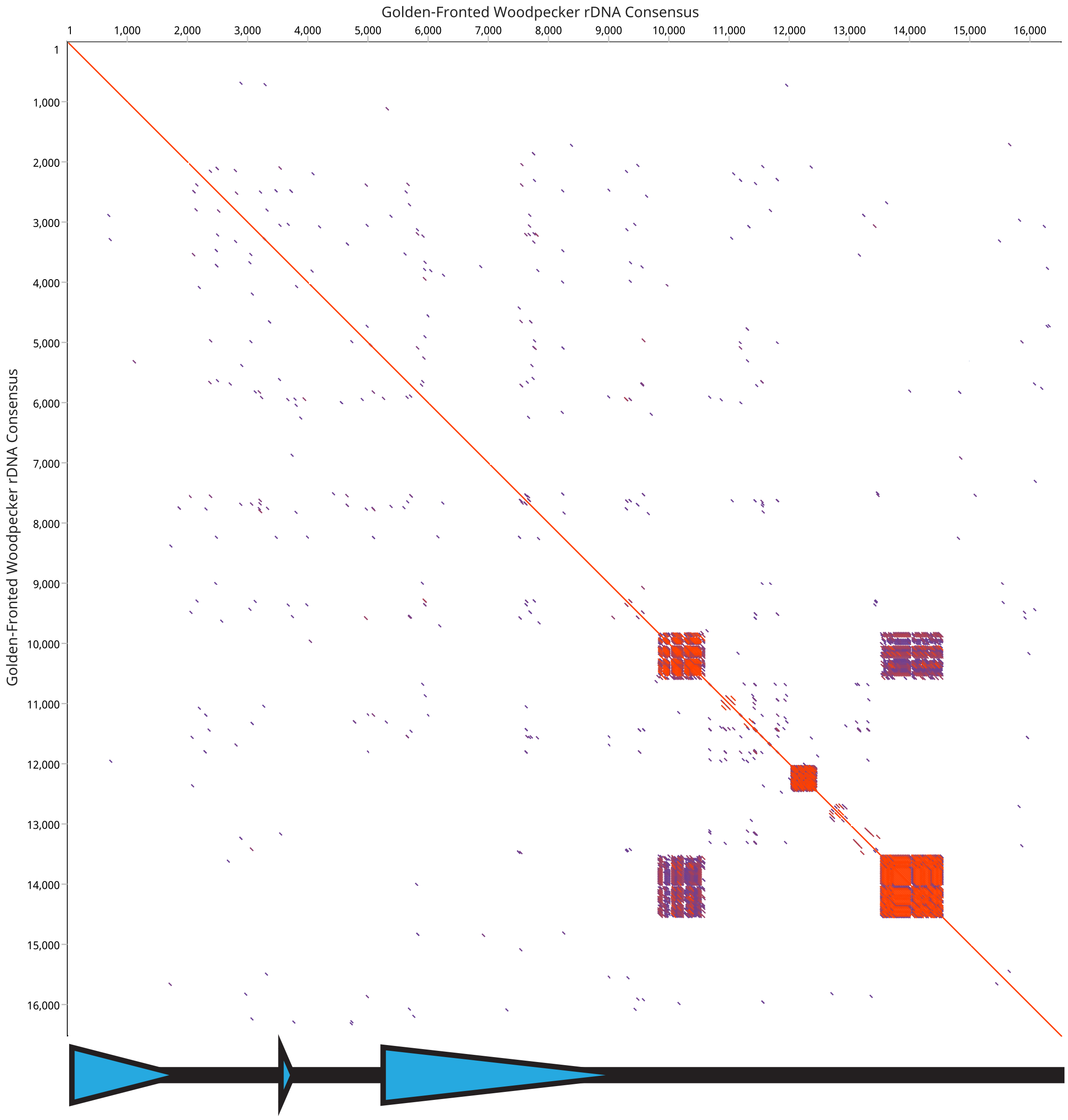

Golden-Fronted Woodpecker rDNA Consensus
1
1,000
2,000
3,000
4,000
5,000
6,000
7,000
8,000
9,000
10,000
11,000
12,000
13,000
14,000
15,000
16,000
Golden-Fronted Woodpecker rDNA Consensus

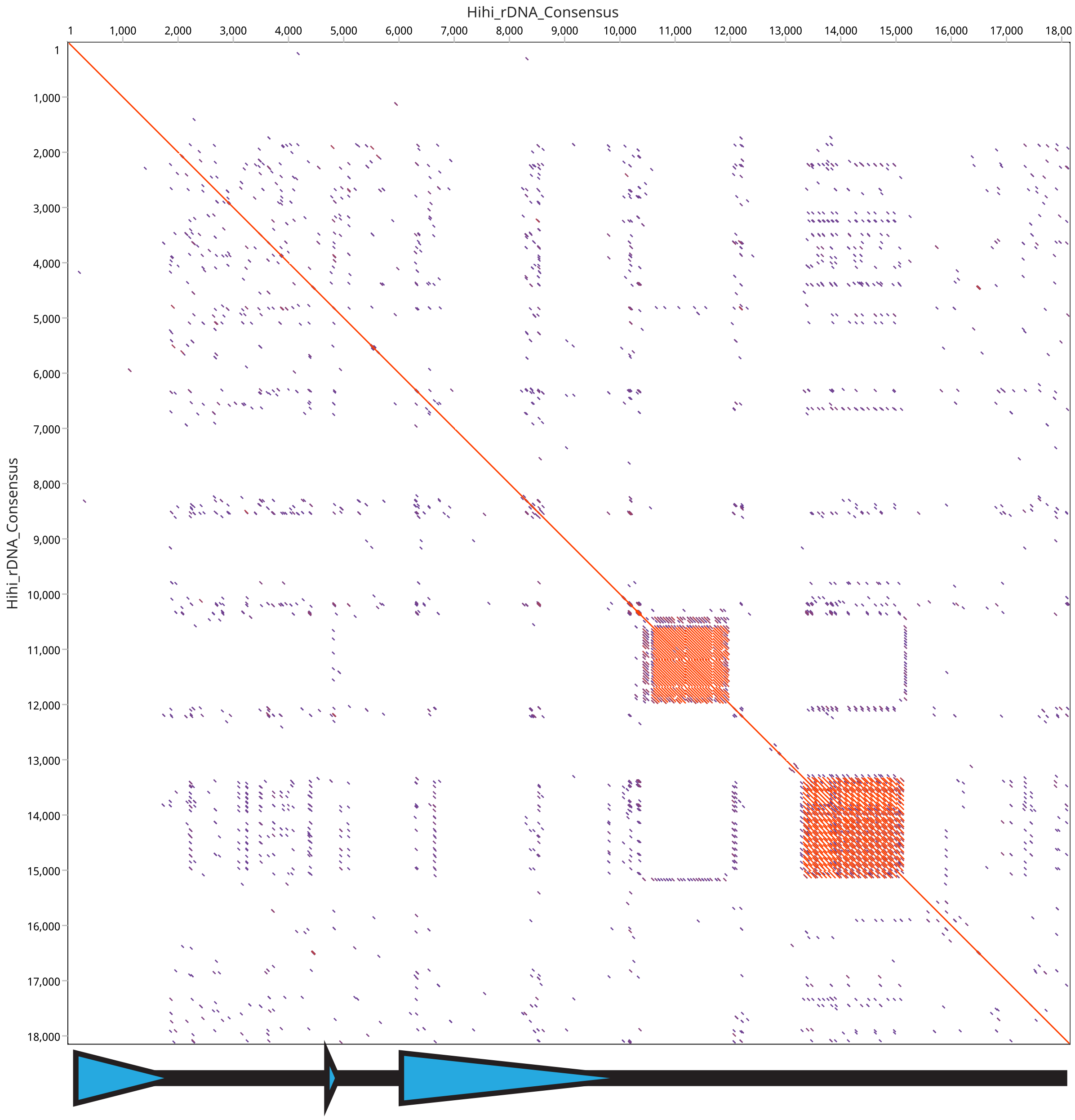

Hihi_rDNA_Consensus
1
1,000
2,000
3,000
4,000
5,000
6,000
7,000
8,000
9,000
10,000
11,000
12,000
13,000
14,000
15,000
16,000
17,000
18,00
Hihi_rDNA_Consensus
1
1,000
2,000
3,000
4,000
5,000
6,000
7,000
8,000
9,000
10,000
11,000
12,000
13,000
14,000
15,000
16,000
17,000
18,000

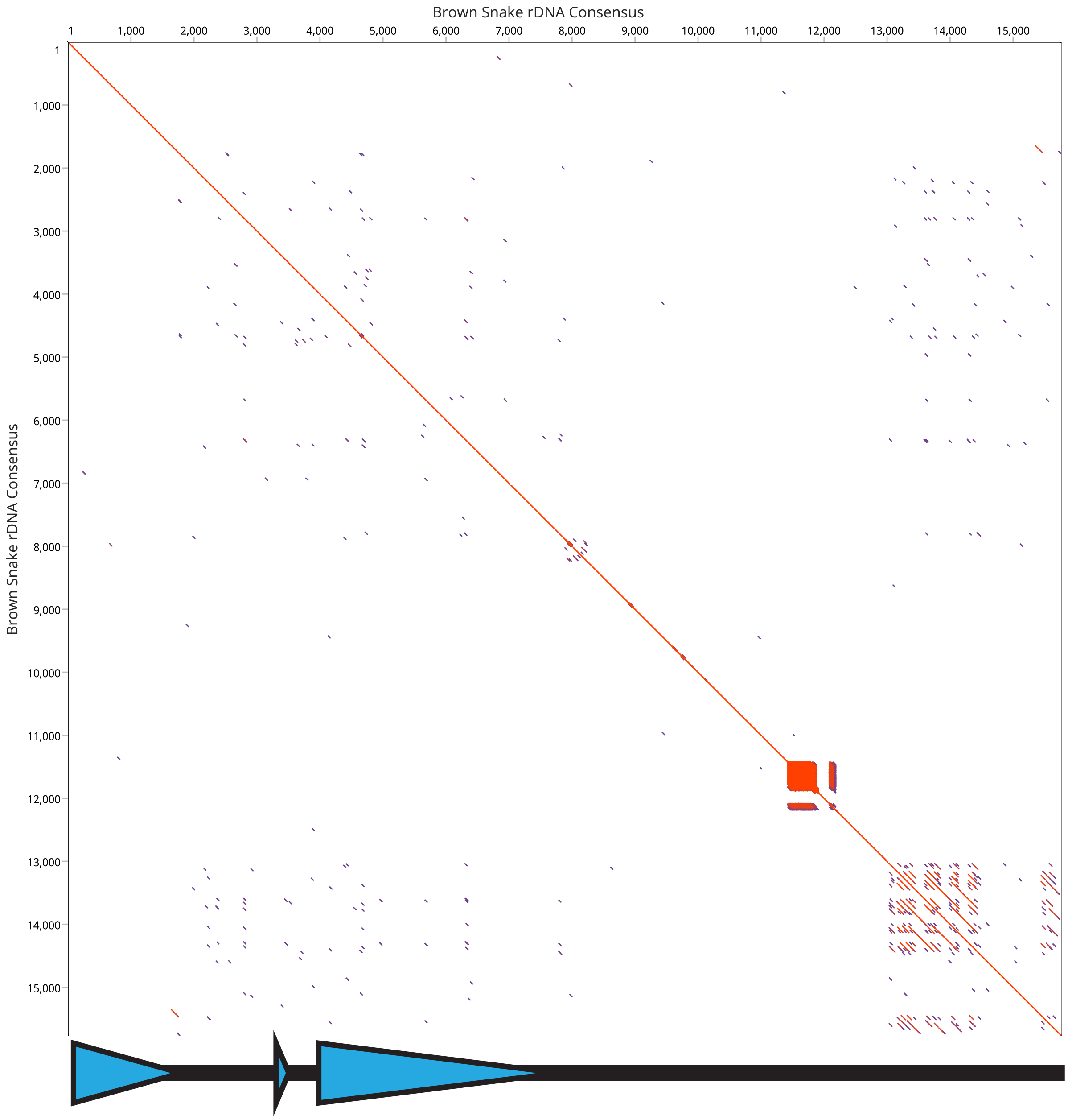
Brown Snake rDNA Consensus
1
1,000
2,000
3,000
4,000
5,000
6,000
7,000
8,000
9,000
10,000
11,000
12,000
13,000
14,000
15,000
Brown Snake rDNA Consensus
1
1,000
2,000
3,000
4,000
5,000
6,000
7,000
8,000
9,000
10,000
11,000
12,000
13,000
14,000
15,000

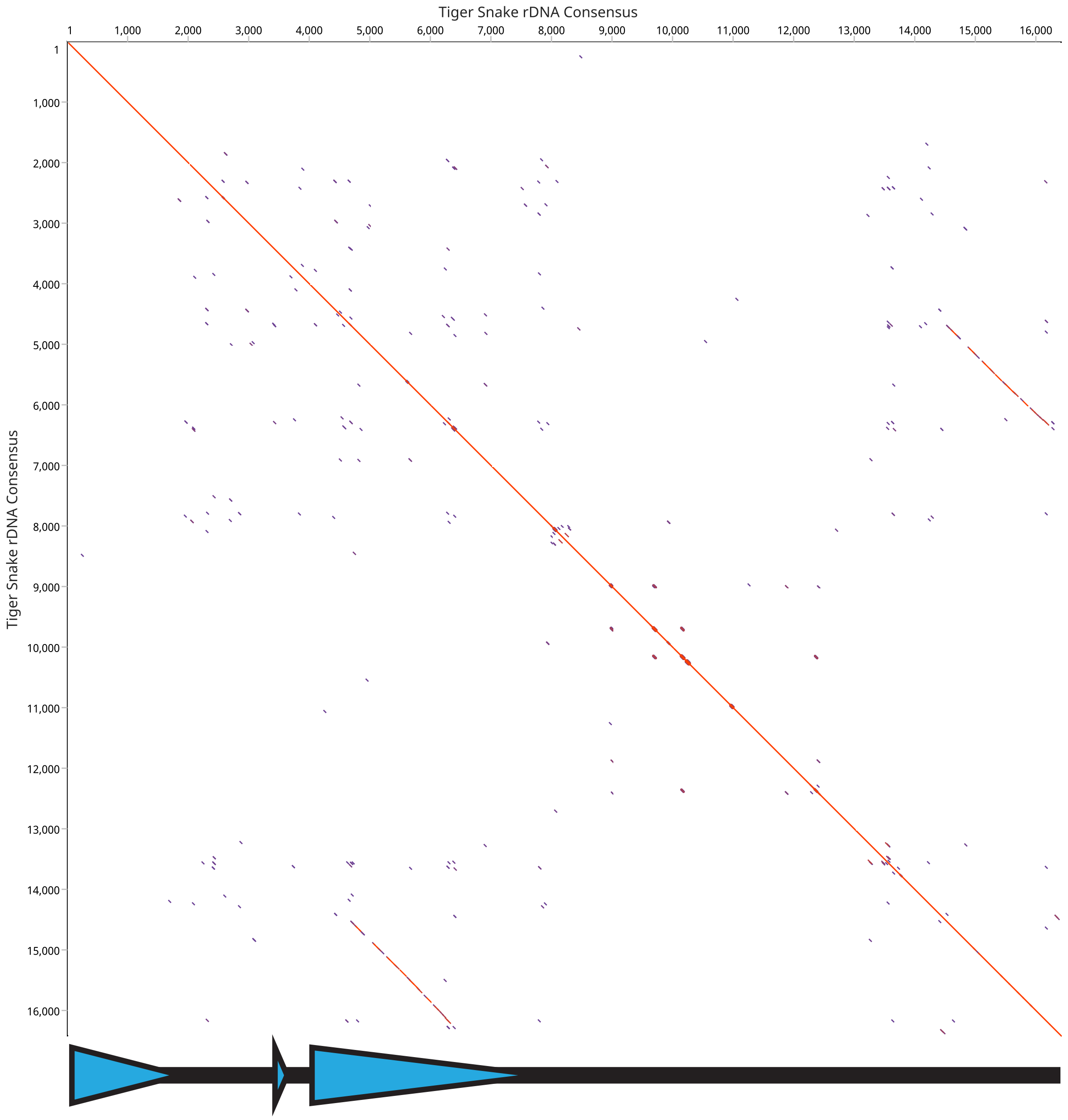
Tiger Snake rDNA Consensus
1
1,000
2,000
3,000
4,000
5,000
6,000
7,000
8,000
9,000
10,000
11,000
12,000
13,000
14,000
15,000
16,000
Tiger Snake rDNA Consensus

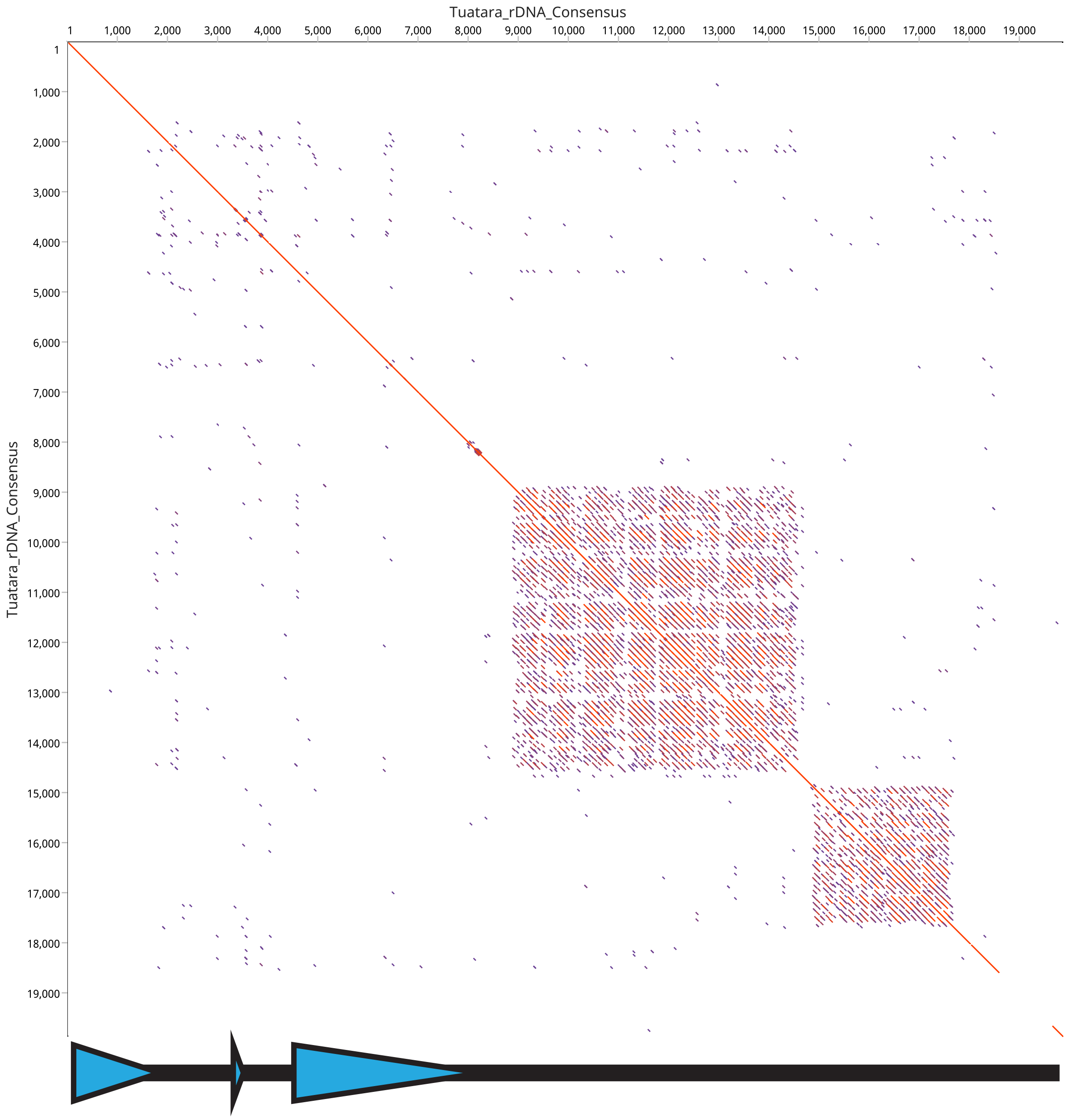
Tuatara_rDNA_Consensus
1
1,000
2,000
3,000
4,000
5,000
6,000
7,000
8,000
9,000
10,000
11,000
12,000
13,000
14,000
15,000
16,000
17,000
18,000
19,000
Tuatara_rDNA_Consensus

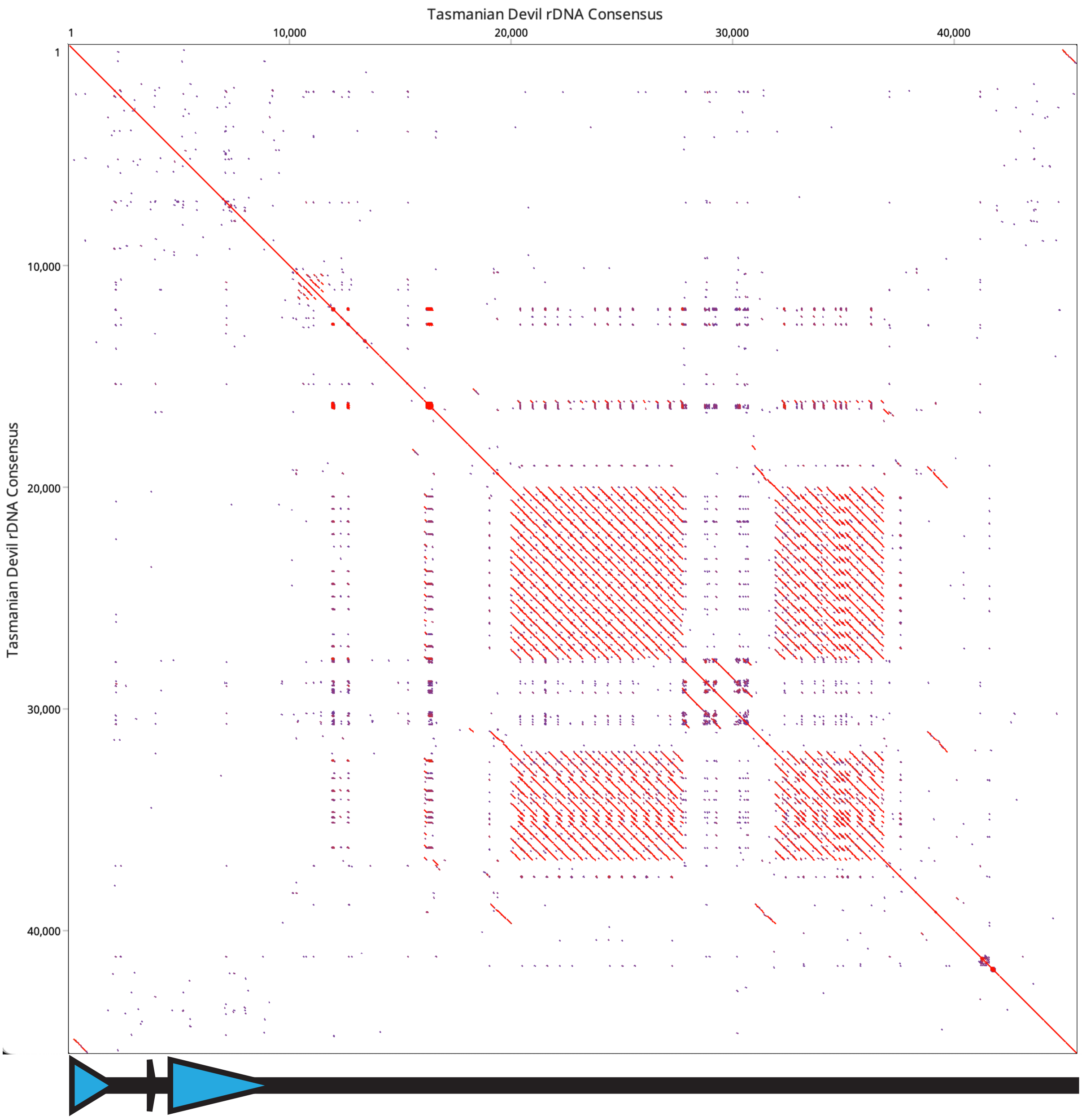

Tasmanian Devil rDNA Consensus
1
10,000
20,000
30,000
40,000
Tasmanian Devil rDNA Consensus
1
10,000
20,000
30,000
40,000

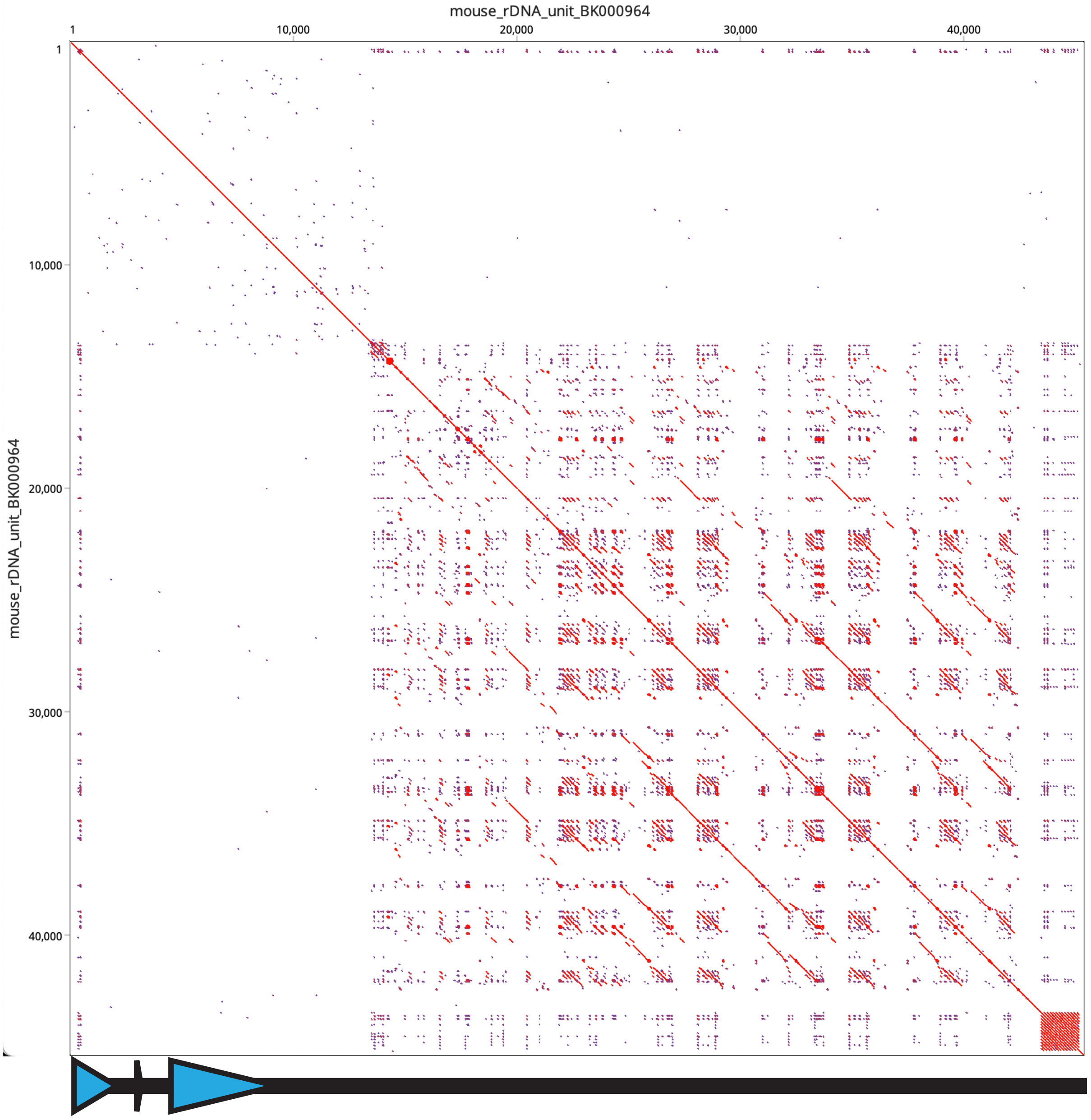
mouse_rDNA_unit_BK000964
1
10,000
20,000
30,000
40,000
mouse_rDNA_unit_BK000964
1
10,000
20,000
30,000
40,000

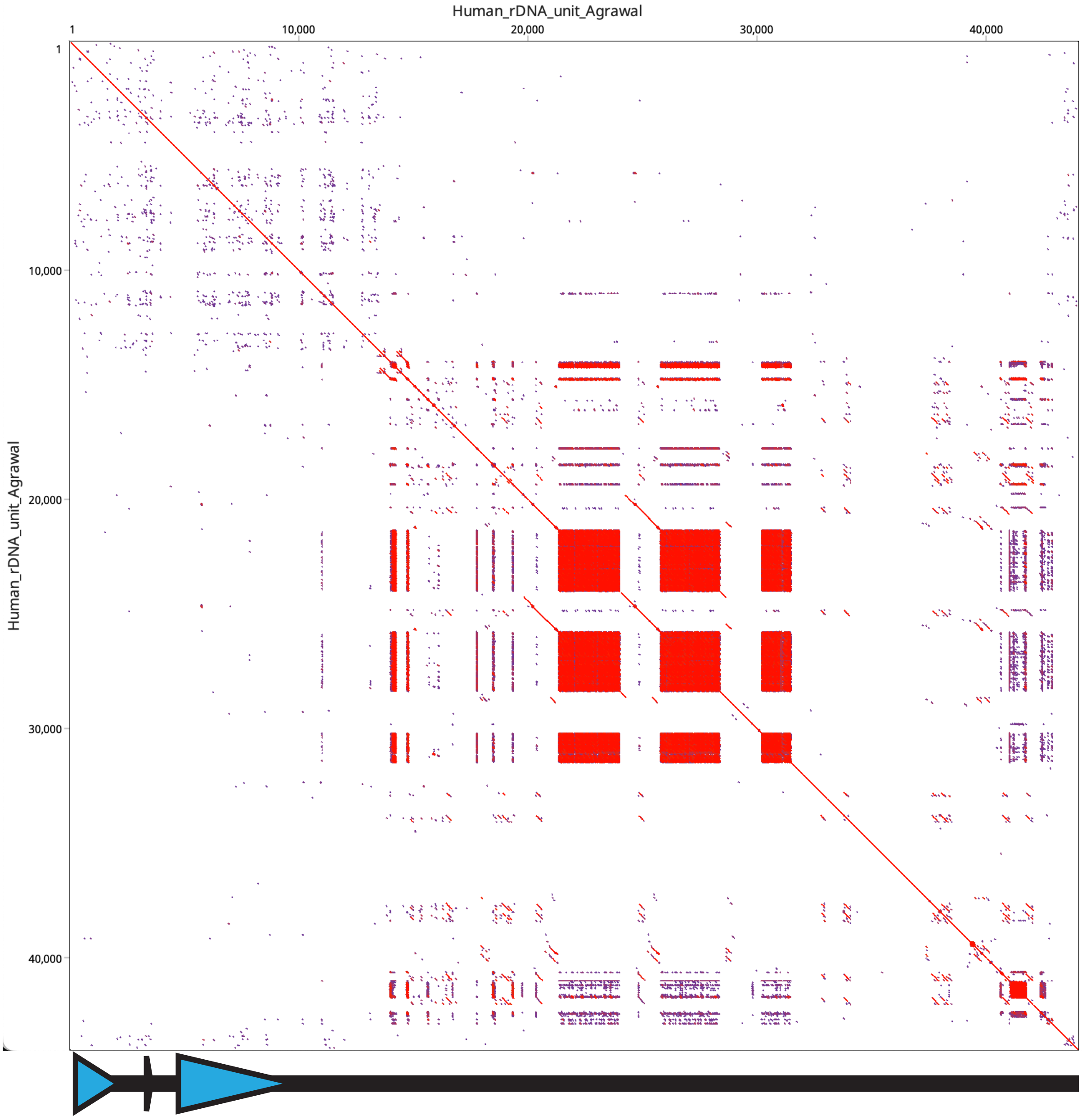

Figure S7 Large dotplots of amniote rDNA units showing presence of tandem sub-repeat arrays. Dotplots are increased-size versions of those shown in **Figure 3**, other than platypus which is shown in **Figure S7**. Names are indicated on the Y axis. Schematics of the rDNA units from **Figure 2** are shown below each dotplot, with blue triangles representing rRNA coding regions. Mouse rDNA unit is NCBI accession BK000964; human rDNA unit is from Agrawal & Ganley, 2018 PLoS One 13: e0207531. Red lines indicate sequence matches in an all-versus-all alignment. Sub-repeat arrays appear as red 'squares' around the diagonal. Microsatellite arrays also appear as red squares, for example in human. Dotplots were created in Geneious (v. 2020.05).
