# Supplementary material for: Origin and maintenance of large ribosomal RNA gene repeat size in mammals
Source: Genetics. 2024 Jul 24;228(1):iyae121. doi: 10.1093/genetics/iyae121 (PMC11373518; doi:10.1093/genetics/iyae121)
Supplement: iyae121_Supplementary_Data [file iyae121_supplementary_data.zip › Figure_S8_GENETICS-2024-307168.pdf]

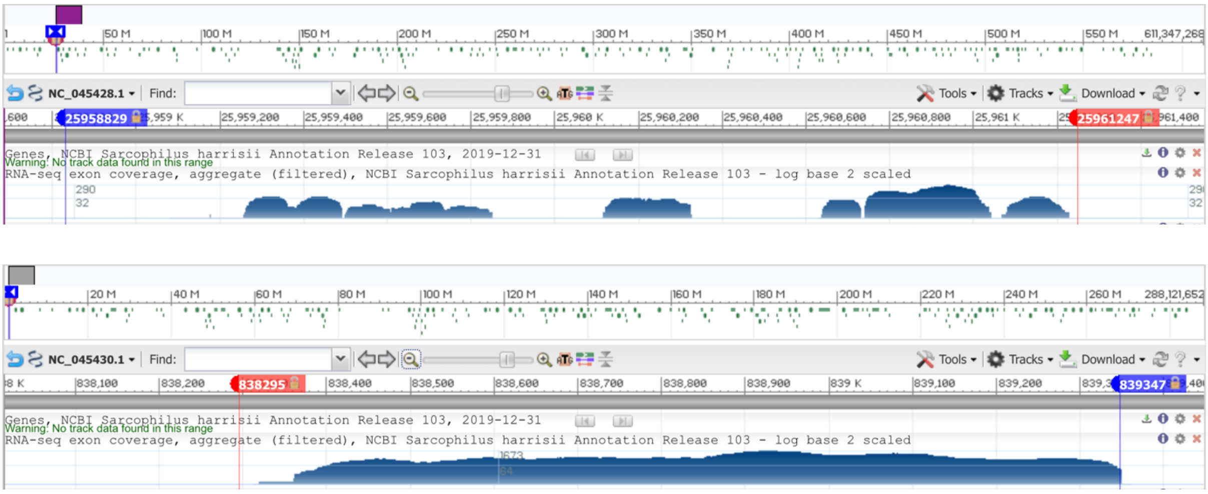

Figure S8. Matches between the Tasmanian devil IGS and the genome. Genome browser track screenshots showing the two matches of the IGS to the Tasmanian devil genome >1 kb identified by BLAST. The start/stop positions of the IGS match are indicated by blue and red flags/lines of the chromosome accession numbers as indicated. In both cases, most of the matching region shows transcriptional signal, but neither region is annotated. The top match is to chromosome 3 and covers 27,648-29,879 of the Tasmanian devil consensus rDNA unit. The bottom match is to chromosome 5 and covers 14,707-15,742 of the Tasmanian devil consensus rDNA unit. The bottom match shows 39% identity to a *Monodelphis domestica* unknown protein (accession XP_056665826) by BLASTx, while the top match does not show similarity to known elements by BLAST or BLASTx. The NCBI genome browser was used.
