# Supplementary material for: Origin and maintenance of large ribosomal RNA gene repeat size in mammals
Source: Genetics. 2024 Jul 24;228(1):iyae121. doi: 10.1093/genetics/iyae121 (PMC11373518; doi:10.1093/genetics/iyae121)
Supplement: iyae121_Supplementary_Data [file iyae121_supplementary_data.zip › Figure_S9_GENETICS-2024-307168.pdf]

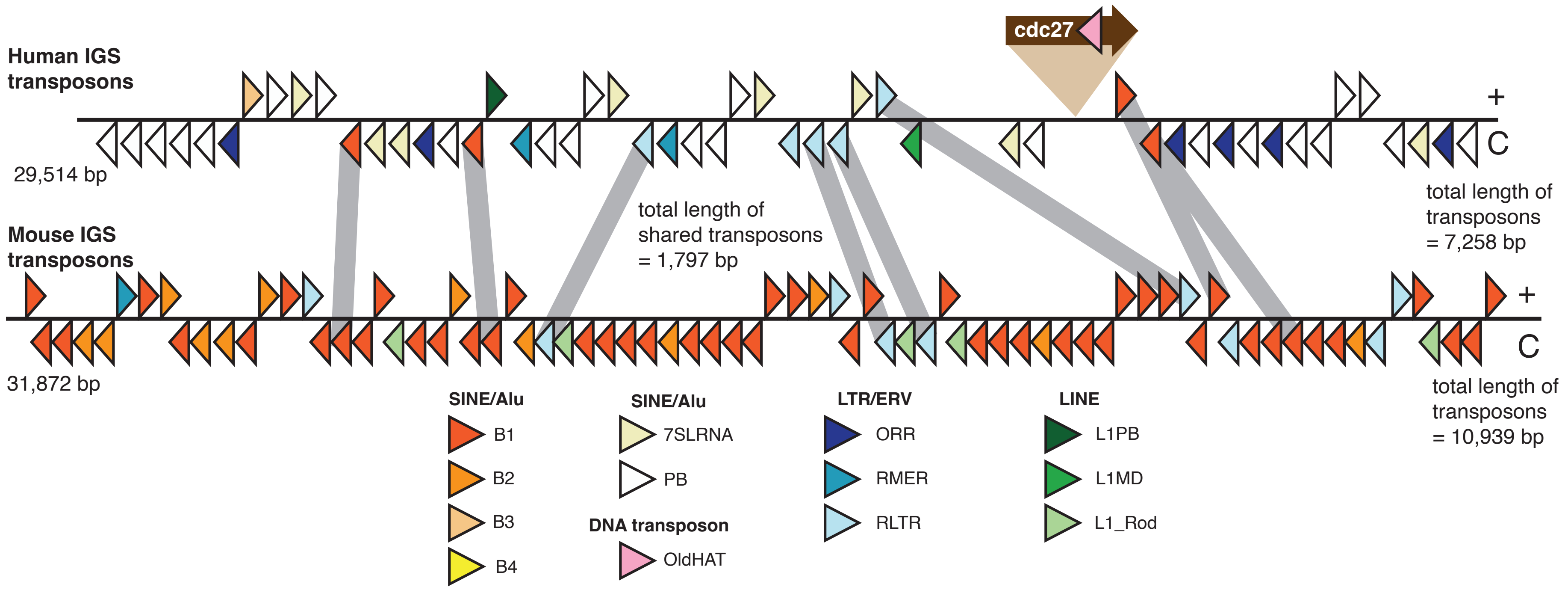

Figure S9. Few IGS TEs are orthologous between human and mouse. TEs detected by Repeatmasker using mouse as source are indicated schematically by triangles, with colors reflecting TE types as indicated below. TE orientation is indicated by triangle direction. Putatively orthologous TEs (determined by conservation of TE type and orientation) are indicated by grey bars. Nested TEs are indicated by internal triangles; and a cdc27 pseudogene found in some primates is illustrated. Total lengths of the IGS, TEs in the IGS, and orthologous TEs are indicated. TE sizing and positioning are not to scale. See **Figure 4** for equivalent figure where human was used as the Repeatmasker source.
