# Supplementary material for: Origin and maintenance of large ribosomal RNA gene repeat size in mammals
Source: Genetics. 2024 Jul 24;228(1):iyae121. doi: 10.1093/genetics/iyae121 (PMC11373518; doi:10.1093/genetics/iyae121)
Supplement: iyae121_Supplementary_Data [file iyae121_supplementary_data.zip › File_S1_GENETICS-2024-307168.pdf]

File S1. Supplemental material description for Macdonald et al, "*Origin and maintenance of large ribosomal RNA gene repeat size in mammals*"

Supplementary Files

Figure S1. Density plots showing that our ONT sequence read BLAST analysis can accurately distinguish 'normal' and 'long' rDNA units, using yeast and human ONT datasets.

Figure S2. Density plots used to determine rDNA unit sizes from ONT sequence reads for all amniote species analyzed in this study other than platypus and Tasmanian devil.

Figure S3. Density plot used to determine rDNA unit sizes from ONT sequence reads for Tasmanian devil, and sequence alignment showing that the short Tasmanian devil rDNA unit lack identifiable similarity to the 5.8S rRNA gene.

Figure S4. Density plot showing that PacBio assembly can accurately determine rDNA unit size using a diamondback terrapin PacBio dataset.

Figure S5. Plot showing the platypus PacBio assembly rDNA unit sizes.

Figure S6. Dotplots showing the sub-repeat arrays present in the platypus ITS1 and IGS, and showing sub-repeat copy number variation between different rDNA units.

Figure S7. Large dotplots of the rDNA units for the amniotes analyzed in this study except platypus showing the presence and absence of sub-repeat arrays in the different species.

Figure S8. Genome browser views showing matches between the Tasmanian devil IGS and the genome that have evidence for transcription.

Figure S9. Schematic showing the TE composition of the human and mouse IGS regions from Repeatmasker using mouse as the source, indicating like Figure 3 that there are few orthologous TEs in the IGSs of these two species.

Figure S10. Alignment details between platypus, mouse, human and Tasmanian devil IGS sequences showing that these sequences are essentially unalignable.

Table S1. Table of published rDNA unit sizes of amniote species.

Table S2. Table showing the sizes of the various parts of the rDNA unit in the amniote species analysed in this study, and what percent of total rDNA unit length these make up.

Table S3. Table of platypus sub-repeat copy numbers and array lengths.

Table S4. Table showing what percent of the different in platypus rDNA unit length is a consequence of variation in sub-repeat copy number.

Table S5. Table showing the proportion of the IGS that is occupied by sub-repeats.

Table S6. Table of the accession numbers of the rDNA units used as the queries in BLAST searches.

<u>Data provided on Figshare (10.17608/k6.auckland.25126664)</u>

For each species analyzed in this study except platypus, there are four files:

- A consensus rDNA unit (in Geneious format)
- The reads used to make the consensus sequence (in fasta format)
- The reads used to calculate rDNA unit sizes (in fasta format)
- Spreadsheet used to calculate rDNA unit sizes (in .xlsx format)

For the platypus, there are six files:

- A consensus rDNA unit (in Geneious format)
- The PacBio reads used for the first PacBio assembly (in fasta format)
- The individual PacBio rDNA units (in Geneious format)
- The three whole rDNA unit-containing contigs from the second PacBio assembly (each in Geneious format)

In addition, the Repeatmasker output file is provided for the rDNA sequence of each species analysed in this study, and for human and mouse rDNA sequences using each species as the Repeatmasker source (text format).
