# Supplementary material for: Origin and maintenance of large ribosomal RNA gene repeat size in mammals
Source: Genetics. 2024 Jul 24;228(1):iyae121. doi: 10.1093/genetics/iyae121 (PMC11373518; doi:10.1093/genetics/iyae121)
Supplement: iyae121_Supplementary_Data [file iyae121_supplementary_data.zip › Table_S1_GENETICS-2024-307168.pdf]

**Table S1. Published rDNA unit sizes of amniote species**

| Species | rDNA unit size (kb) | Reference(s)[a] |
|---|---|---|
| *Homo sapiens* (human) | 44.0 | (GONZALEZ AND SYLVESTER 1995; AGRAWAL AND GANLEY 2018) |
| *Mus musculus* (mouse) | 45.3 | (GROZDANOV *et al.* 2003) |
| *Pan troglodytes* (chimpanzee) | 41.8 | (AGRAWAL AND GANLEY 2018) |
| *Gorilla gorilla* (gorilla) | 37.6 | |
| *Pongo abelii* (orangutan) | 40.9 | |
| *Normascus leucogenys* (gibbon) | 42.9 | |
| *Macaca mulatta* (rhesus macaque) | 41.7 | |
| *Callithrix jacchus* (common marmoset) | 39.6 | |
| *Apodemus* sp. (field mice) | ~ 40 | (SUZUKI *et al.* 1990) |
| *Rattus rattus* (rat) | 35.5 - 39.9 | (STUMPH *et al.* 1979; BRAGA *et al.* 1982) |
| *Bos taurus* (cow) | ~ 34 | (MEUNIER-ROTIVAL *et al.* 1979) |
| *Meles meles* (badger) | 42.8 | Annotation on unplaced contig; NCBI accession NW_025721128.1; (HOSODA *et al.* 1993) |
| *Canis familiaris* (domestic dog) | ~ 40 | (HOSODA *et al.* 1993) |
| *Vulpes vulpes* (red fox) | | |
| *Nyctereutes procyonoides* (Japanese raccoon-dog) | | |

| | | |
|---|---|---|
| *Mustela* sp. (weasel) | | |
| *Mustela putorius* (domestic ferret) | | |
| *Martes melampus* (Japanese marten) | | |
| *Gallus gallus* (chicken) | 11-50 | (Delany and Krupkin 1999; Dyomin *et al.* 2019) |
| *Darevskia armeniaca* (Armenian lizard) | ~ 15 | (Voronov *et al.* 2008) |
| *Darevskia raddei* (Azerbaidhan lizard) | ~ 15 | |
| *Darevskia valentine* (Caucasian rock lizard) | ~ 15 | |
| *Lacerta agilis* (sand lizard) | ~ 12 | |
| *Zootoca vivipara* (viviparous lizard) | ~ 12 | |
| *Eulamprus murrayi* (blue-speckled forest skink) | ~ 12 | |
| *Varanus exanthematicus* (monitor lizard) | ~ 11 | |
| *Iguana iguana* (green iguana) | ~ 11 | |
| *Uta stansburiana* (common side-blotched lizard) | ~ 15 | |
| *Laudakia caucasia* (Caucasian agama) | ~ 10 | |
| *Tarentola mauritanica* (common wall gecko) | 15.2 - 15.6 | (Cortadas and Pavon 1982) |
| *Natrix maura* (viperine water snake) | 15.3 -15.4 | |
| *Bothrops neuwiedi* (Neuwied's lancehead) | 11.1 | |
| *Dromicus poecilogyrus* (cuban racer) | 14.2 | |
| *Testudo graeca* (spur-thighed tortoise) | 19.3 | |

## a References


Agrawal, S., and A. R. D. Ganley, 2018 The conservation landscape of the human ribosomal RNA gene repeats. PLoS One 13**:** e0207531.

Braga, E. A., T. N. Yussifov and V. V. Nosikov, 1982 Structural organization of rat ribosomal genes restriction endonuclease analysis of genomic and cloned ribosomal DNAs. Gene 20**:** 145-156.

Cortadas, J., and M. C. Pavon, 1982 The organization of ribosomal genes in vertebrates. EMBO J. 1**:** 1075-1080.

Delany, M. E., and A. B. Krupkin, 1999 Molecular characterization of ribosomal gene variation within and among NORs segregating in specialized populations of chicken. Genome 42**:** 60-71.

Dyomin, A., S. Galkina, V. Fillon, S. Cauet, C. Lopez-Roques *et al.*, 2019 Structure of the intergenic spacers in chicken ribosomal DNA. Genet. Sel. Evol. 51**:** 59.

Gonzalez, I. L., and J. E. Sylvester, 1995 Complete sequence of the 43-kb human ribosomal DNA repeat: analysis of the intergenic spacer. Genomics 27**:** 320-328.

Grozdanov, P., O. Georgiev and L. Karagyozov, 2003 Complete sequence of the 45-kb mouse ribosomal DNA repeat: analysis of the intergenic spacer. Genomics 82**:** 637-643.

Hosoda, T., H. Suzuki, T. Yamada and K. Tsuchiya, 1993 Restriction site polymorphism in the ribosomal DNA of eight species of Canidae and Mustelidae. Cytologia 58**:** 223-230.

Meunier-Rotival, M., J. Cortadas, G. Macaya and G. Bernardi, 1979 Isolation and organization of calf ribosomal DNA. Nucleic Acids Res. 6**:** 2109-2123.

Stumph, W. E., J. R. Wu and J. Bonner, 1979 Determination of the size of rat ribosomal deoxyribonucleic acid repeating units by electron microscopy. Biochemistry 18**:** 2864-2871.

Suzuki, H., K. Tsuchiya, M. Sakaizumi, S. Wakana, O. Gotoh *et al.*, 1990 Differentiation of restriction sites in ribosomal DNA in the genus *Apodemus*. Biochem. Genet. 28**:** 137-149.

Voronov, A. S., D. V. Shibalev and N. S. Kupriianova, 2008 Specific organization of ribosomal DNA arrays in Squamata. Genetika 44**:** 1547-1552.
