# Supplementary material for: Origin and maintenance of large ribosomal RNA gene repeat size in mammals
Source: Genetics. 2024 Jul 24;228(1):iyae121. doi: 10.1093/genetics/iyae121 (PMC11373518; doi:10.1093/genetics/iyae121)
Supplement: iyae121_Supplementary_Data [file iyae121_supplementary_data.zip › Table_S3_GENETICS-2024-307168.pdf]

**Table S3. Platypus rDNA sub-repeat copy numbers**

| rDNA unit | ITS1 610 bp sub-repeat number | IGS 220 bp sub-repeat number | IGS 105 bp sub-repeat number | IGS 585 bp sub-repeat number | Total sub-repeat length (bp) | rDNA unit length (bp) |
|---|---|---|---|---|---|---|
| 1st PacBio assembly unit | 7 | 24 | 7 | 18 | 21,351 | 38,785 |
| PacBio contig2 unit 1 | 8 | 10 | 7 | 5 | 11,932 | 30,337 |
| PacBio contig2 unit 2 | 6 | 13 | 7 | 16 | 17,327 | 35,763 |
| PacBio contig2 unit 3 | 7 | 32 | 7 | 14 | 21,295 | 40,427 |
| PacBio contig2 unit 4 | 7 | 11 | 7 | 14 | 16,256 | 34,612 |
| PacBio contig40 unit 1 | 11 | 14 | 7 | 16 | 21,519 | 39,649 |
| PacBio contig40 unit 2 | 7 | 24 | 7 | 9 | 15,789 | 33,791 |
| PacBio contig40 unit 3 | 7 | 13 | 7 | 16 | 18,056 | 35,954 |
| PacBio contig85 unit 1 | 7 | 19 | 7 | 14 | 17,299 | 34,996 |
