# Supplementary material for: Origin and maintenance of large ribosomal RNA gene repeat size in mammals
Source: Genetics. 2024 Jul 24;228(1):iyae121. doi: 10.1093/genetics/iyae121 (PMC11373518; doi:10.1093/genetics/iyae121)
Supplement: iyae121_Supplementary_Data [file iyae121_supplementary_data.zip › Table_S6_GENETICS-2024-307168.pdf]

**Table S6. Query rDNA units used to perform BLAST searches**

| Query rDNA unit species of origin | Genbank accession number | Species searched against |
|---|---|---|
| *Saccharomyces cerevisiae* (budding yeast) | U53879.1 (RDN37; Chr XII, 2217 .. 31308) | *Saccharomyces cerevisiae* (budding yeast) |
| *Homo sapiens* (human) | GL000220 | *Homo sapiens* (human) |
| | | *Sarcophilus harrisii* (Tasmanian devil) |
| | | *Ornithorhynchus anatinus* (platypus) |
| | | *Pan troglodytes* (chimpanzee) |
| *Xenopus laevis* (African clawed frog) | X02995.1 | *Malaclemys terrapin* (diamondback turtle) |
| | | *Notechis scutatus* (tiger snake) |
| | | *Pseudonaja textilis* (brown snake) |
| | | *Rhinella marina* (cane toad) |
| | | *Sphenodon punctatus* (Tuatara) |
| *Gallus gallus* (domestic chicken) | KT445934 | *Melanerpes aurifrons* (golden-fronted woodpecker) |
| | | *Notiomystis cincta* (stitchbird hihi) |
| *Pan troglodytes* (chimpanzee) | KX061886 | *Pan troglodytes* (chimpanzee) |
